# TWIST1^+^FAP^+^ fibroblasts in the pathogenesis of intestinal fibrosis in Crohn’s disease

**DOI:** 10.1172/JCI179472

**Published:** 2024-07-18

**Authors:** Yao Zhang, Jiaxin Wang, Hongxiang Sun, Zhenzhen Xun, Zirui He, Yizhou Zhao, Jingjing Qi, Sishen Sun, Qidi Yang, Yubei Gu, Ling Zhang, Chunhua Zhou, Youqiong Ye, Ningbo Wu, Duowu Zou, Bing Su

**Affiliations:** 1 Department of Gastroenterology, Center for Immune-Related Diseases, Ruijin Hospital,; 2 Shanghai Institute of Immunology, Department of Immunology and Microbiology, and Ministry of Education Key Laboratory of Cell Death and Differentiation, and; 3 Department of General Surgery, Ruijin Hospital, Shanghai Jiao Tong University School of Medicine, Shanghai, China.

**Keywords:** Gastroenterology, Extracellular matrix, Fibrosis, Inflammatory bowel disease

## Abstract

Intestinal fibrosis, a severe complication of Crohn’s disease (CD), is characterized by excessive extracellular matrix (ECM) deposition and induces intestinal strictures, but there are no effective antifibrosis drugs available for clinical application. We performed single-cell RNA sequencing (scRNA-Seq) of fibrotic and nonfibrotic ileal tissues from patients with CD with intestinal obstruction. Analysis revealed mesenchymal stromal cells (MSCs) as the major producers of ECM and the increased infiltration of its subset FAP^+^ fibroblasts in fibrotic sites, which was confirmed by immunofluorescence and flow cytometry. Single-cell transcriptomic profiling of chronic dextran sulfate sodium salt murine colitis model revealed that CD81^+^Pi16^–^ fibroblasts exhibited transcriptomic and functional similarities to human FAP^+^ fibroblasts. Consistently, FAP^+^ fibroblasts were identified as the key subtype with the highest level of ECM production in fibrotic intestines. Furthermore, specific knockout or pharmacological inhibition of TWIST1, which was highly expressed by FAP^+^ fibroblasts, could significantly ameliorate fibrosis in mice. In addition, TWIST1 expression was induced by CXCL9^+^ macrophages enriched in fibrotic tissues via IL-1β and TGF-β signal. These findings suggest the inhibition of TWIST1 as a promising strategy for CD fibrosis treatment.

## Abstract 2

*FAP*^+^ fibroblasts are identified as a key subtype responsible for pathogenic ECM production in Crohn’s disease-associated intestinal fibrosis.

## Graphical abstract

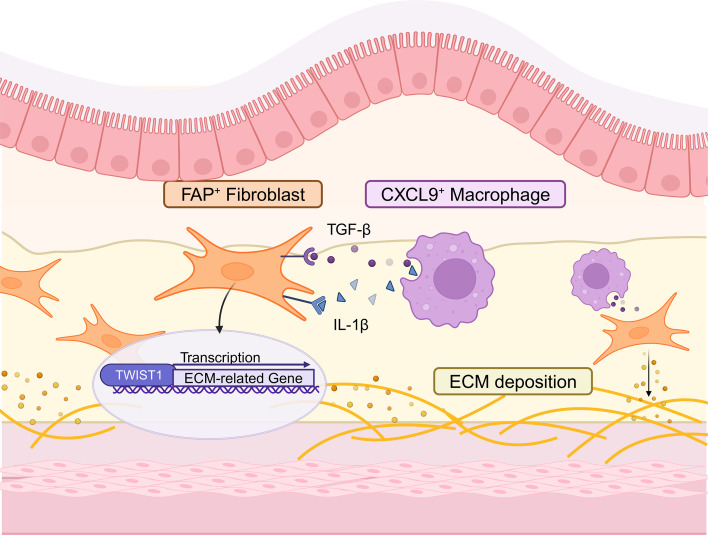

## Introduction

Crohn’s disease (CD) is a chronic gastrointestinal inflammatory disease with limited effective pharmacological treatments and several clinical complications like strictures and fistulas. Approximately 50% of patients with CD develop intestinal fibrosis characterized by intestinal strictures (1). Most patients with fibrosis require surgical intervention, and yet half of them relapse within 10 years (2). Several therapeutic targets have been discovered for antifibrosis treatment, including proinflammatory cytokines (TNF-α, IL-17, IL-36, and others), TGF-β pathways, matrix metalloproteinases, and other pertinent molecular pathways (3, 4). However, no specific antifibrotic therapy has been approved by the FDA to date (5, 6), as the specific mechanism of intestinal fibrosis is still not fully understood.

Diverse cell types (epithelial, stromal, immune, etc.) and their reciprocal communication play important roles in intestinal homeostasis maintenance or disease pathogenesis (7, 8). The advancement of sequencing technologies has enabled us to establish a comprehensive single-cell-level resolution of the heterogeneity and regulatory networks of various cell types in normal and pathological states (9). Multiple studies have been performed to unveil the cellular and molecular mechanisms of CD. CD39^+^ Th17 cells were reported as an enriched population in CD (10), and a unique cellular module containing immune and stromal cells was identified as novel biomarkers of treatment response to anti-TNF therapy (11). However, the cellular heterogeneity of the intestinal fibrosis, which is one of the key features of CD has not been thoroughly investigated.

Fibrosis is caused by persistent abnormal activation of stromal cells, resulting in the excessive accumulation of extracellular matrix (ECM) (12, 13). Myofibroblasts marked by α-SMA were previously considered to be the main activated form of stromal cells that produce ECM (14). With the advancement of single-cell genomics analyses and the development of novel genetic models, several subsets of fibroblasts are now considered to be the major ECM producers in multiple organs, such as lung, kidney, and skin (15–17). However, the dominant fibroblast subtypes responsible for excessive ECM deposition in intestinal fibrosis remain unclear. Therefore, identifying the key subtype of stromal contributors in intestinal fibrosis is pivotal for an in-depth understanding of pathogenesis, which could then facilitate the development of novel antifibrotic treatments.

In this study, we utilized surgical samples from patients with CD to perform single-cell RNA sequencing (scRNA-Seq) and unveiled the heterogeneity of stromal cells in the CD microenvironment. We aimed to identify the key fibroblast subtype responsible for ECM production and investigate its activation mechanism in intestinal fibrosis for the development of potential strategies to resolve intestinal fibrosis in patients with CD.

## Results

### Single-cell transcriptome analysis reveals the landscape of intestinal fibrosis in patients with CD.

**Figure 1:**
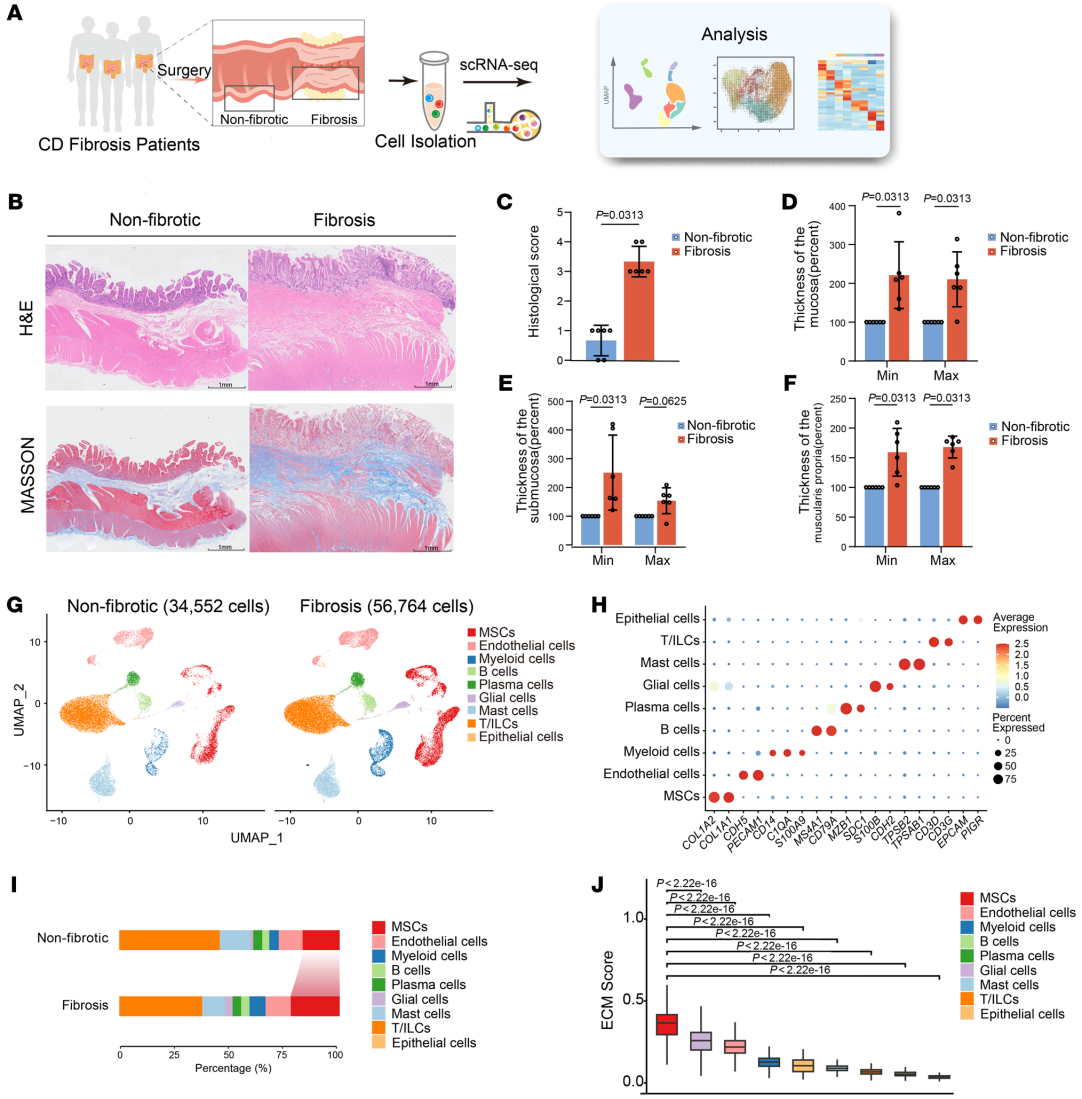
Cellular landscape of fibrotic and nonfibrotic tissues from patients with intestinal fibrosis. (**A**) Graphic overview of the study design. Surgical specimens of fibrotic and adjacent nonfibrotic intestinal segments from patients with CD were processed into single-cell suspensions and subjected to scRNA-Seq using 10x Genomics. Integrated analyses of single-cell transcriptome data are shown in the rectangle to the right. (**B**) Representative plots of H&E and Masson’s trichrome staining of fibrotic and nonfibrotic intestine tissues from a patient with CD. Scale bar: 1 mm. (**C**) Bar plots showing histologic scores of the fibrotic intestinal (*n* = 6) and nonfibrotic (*n* = 6) segments. Data represent the mean ± SD. Statistical differences were determined by paired Wilcoxon’s rank-sum tests. (**D**–**F**) The relative minimal (min) and maximal (max) width of the mucosa (**D**), submucosa (**E**), and muscularis propria (**F**) of the fibrotic and nonfibrotic intestine. All nonfibrotic values were normalized to 100% to calculate the relative thickness of the fibrosis site. Data represent the mean ± SD. Statistical differences were determined by paired Wilcoxon’s rank-sum tests. (**G**) Uniform manifold approximation and projection (UMAP) plots showing 9 major cell types from 6 fibrotic samples (56,764 cells) and 6 nonfibrotic samples (34,552 cells). (**H**) Dot plots of representative markers in the indicated major cell types. The average gene expression and percentage of cells expressed are shown by dot color and size, respectively. (**I**) Bar graph showing the percentage of major cell types in fibrotic and nonfibrotic samples. (**J**) Box plots showing the ECM signature score of each cell type in fibrotic states. Statistical differences were determined by 1-way ANOVA with Bonferroni’s correction.

To investigate the cellular landscape of intestinal fibrosis and the associated molecular characteristics, we collected fibrotic and nonfibrotic surgical specimens from the ilea of patients with CD who underwent intestinal resection for fibrotic stricture (Figure 1A). In comparison with that at nonfibrotic sites, the ileum at the fibrotic site was characterized by narrower lumens and thicker intestinal wall (Supplemental Figure 1A; supplemental material available online with this article; https://doi.org/10.1172/JCI179472DS1). H&E and Masson’s trichrome staining of histological sections revealed more collagen deposition and higher histological scores in the fibrotic tissue area than in the nonfibrotic tissue area (Figure 1, B and C). The thickness of the entire intestinal tissue of fibrotic sites had increased, including the mucosa, submucosa, and muscularis propria (Figure 1, D–F).

Single-cell transcriptomic sequencing was performed on a 10x Genomics platform. After quality control and doublets removal, we retained a total of 91,316 high-quality cells, including 56,764 cells from 6 fibrotic tissue samples and 34,552 cells from 6 nonfibrotic tissues. We identified 9 main cell types through unsupervised clustering and classical marker gene annotation, including mesenchymal stroma cells (MSCs), endothelial cells, myeloid cells, B cells, plasma cells, enteric glial cells, mast cells, T cells or innate lymphoid cells (T/ILCs), and epithelial cells (Figure 1, G and H, and Supplemental Figure 1, B and C). By further analysis of the frequency of cell populations within each group, we observed an increase in the abundance of enteric glial cells and an increased trend of MSC and myeloid cell abundance but a decrease in the abundance of T/ILCs and mast cells in fibrotic tissues, suggesting the key role of MSCs and myeloid cells in the pathological process of fibrosis (Figure 1I and Supplemental Figure 1, D and E).

Excessive deposition of ECM is the core pathological feature of fibrosis. To reveal the specific cell type contributing to excessive ECM deposition, we calculated the ECM gene signature score to assess the ECM-producing capacity of each cell type. In detail, we utilized the genes of Extracellular Matrix Organization from Gene Ontology Resource (https://www.ebi.ac.uk/QuickGO/term/GO:0030198) to calculate the average expression of these genes for each cell type as the ECM signature score. We also referred to previous literature and selected the gene sets of collagen, glycoprotein, and proteoglycan from it; then we calculated the signature score using the methods above (16). We found that MSCs exhibited the highest ECM score in fibrotic areas (Figure 1J and Supplemental Figure 1F), highlighting the pivotal role of MSCs in ECM production during intestinal fibrosis. Overall, single-cell transcriptome data indicated a fibrosis-specific cell landscape and emphasized the critical involvement of MSCs in ECM production and fibrotic pathogenesis.

### Cellular heterogeneity of mesenchymal stromal cells from fibrotic intestine tissues.

**Figure 2:**
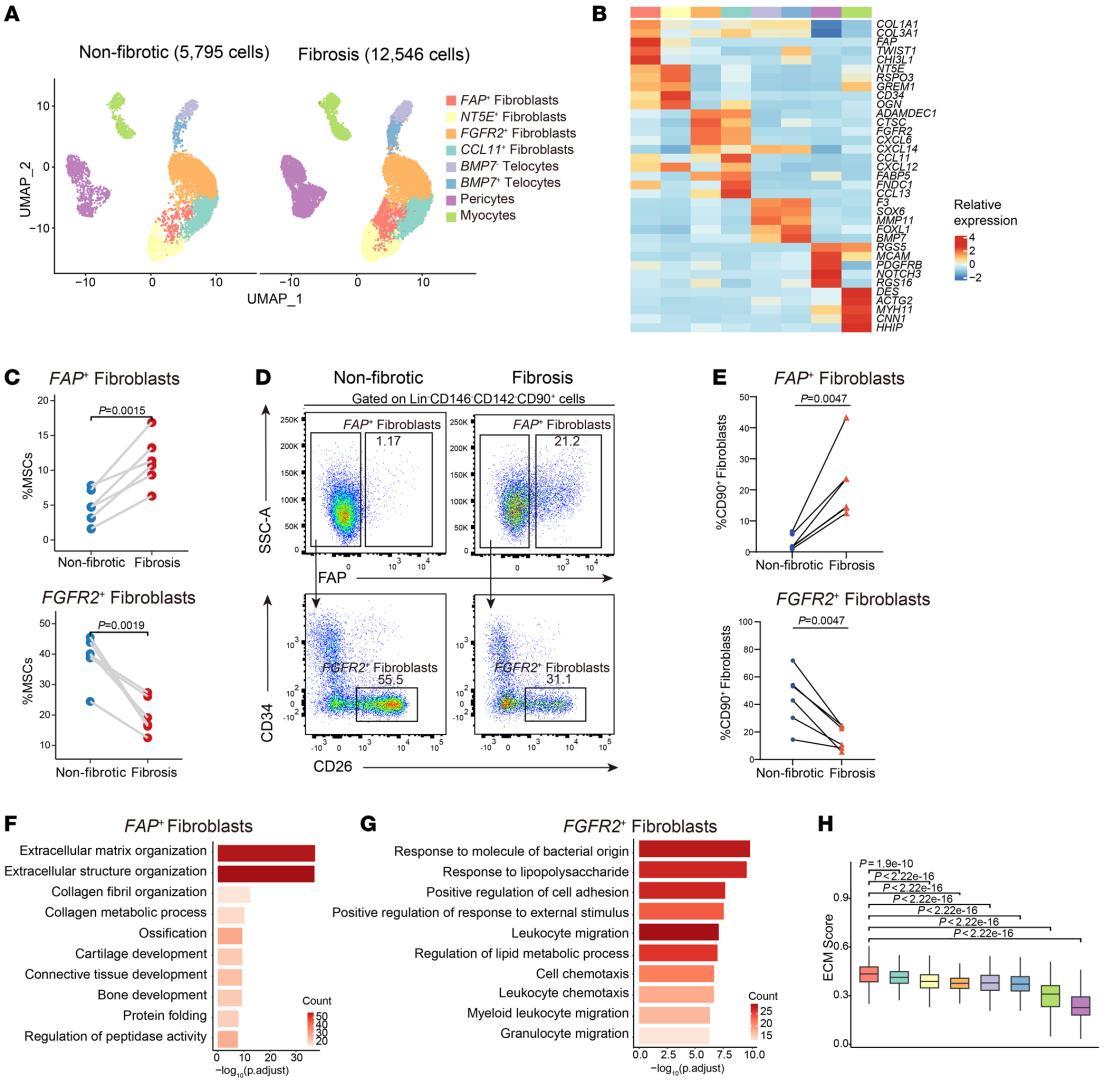
Heterogeneity of mesenchymal stromal cells in intestinal fibrosis. (**A**) UMAP plots of subclustered mesenchymal stromal cells in nonfibrotic and fibrotic states. (**B**) Heatmap showing the relative expression (*Z* score) of representative markers in each MSC subtype. Clusters are colored as in **A**. (**C**) Comparison of frequencies of FAP^+^ fibroblasts and FGFR2^+^ fibroblasts of MSCs in paired fibrotic intestinal samples (*n* = 6) and nonfibrotic intestinal samples (*n* = 6). Statistical differences were determined by paired *t* tests. (**D**) Representative flow cytometry plots of FAP^+^ fibroblasts (top) and FGFR2^+^ fibroblasts (bottom) in fibrotic and nonfibrotic mucosa samples. The gating strategies for MSCs are shown in Supplemental Figure 2D. (**E**) Flow cytometry analysis revealed the proportional variation in FAP^+^ fibroblasts and FGFR2^+^ fibroblasts to CD90^+^ fibroblasts in fibrotic and nonfibrotic sites. The points corresponding to the paired samples (*n* = 6) in the graph are connected. Statistical differences were determined by paired *t* tests. (**F** and **G**) Representative Gene Ontology (GO) enrichment of the marker genes expressed in FAP^+^ fibroblasts (**F**) and FGFR2^+^ fibroblasts (**G**). A hypergeometric test was performed with FDR-adjusted *P* values. (**H**) Box plots showing the ECM signature score of each subcluster of MSCs in fibrotic states. Statistical differences were determined by 1-way ANOVA with Bonferroni’s correction.

To decipher the heterogeneity of MSCs in intestinal fibrosis and identify specific fibrosis-driving MSC populations, we performed subclustering of MSCs and found 4 major subsets: fibroblasts, telocytes, pericytes, and myocytes based on their expression profile (Figure 2A and Supplemental Figure 2A). Telocytes can be divided into 2 populations based on BMP7 expression level. We identified 4 subsets of fibroblasts by clustering analysis, NT5E^+^ fibroblasts, FAP^+^ fibroblasts, CCL11^+^ fibroblasts, and FGFR2^+^ fibroblasts, which were annotated by specific marker gene expression (Figure 2, A and B). We found that the abundance of FAP^+^ fibroblasts (*P* = 0.0015) and NT5E^+^ fibroblasts (*P* = 0.049) was notably increased within fibrotic areas, while FGFR2^+^ fibroblasts (*P* = 0.0019) were more abundant in nonfibrotic areas (Figure 2C and Supplemental Figure 2, B and C).

Flow cytometry experiments further validated the compositions of different stromal cell subsets in fibrotic and nonfibrotic areas. We analyzed populations of pericytes, myocytes, telocytes, FAP^+^ fibroblasts, and FGFR2^+^ fibroblasts from fibrotic and nonfibrotic areas. Detailed gating strategy was based on the expression of stromal cell subtype markers (Supplemental Figure 2, D and E). Consistent with the sequencing data, the abundance of FAP^+^ fibroblast was significantly increased in fibrotic intestine samples (*P* = 0.0047), while FGFR2^+^ fibroblast abundance exhibited a notable reduction (*P* = 0.0047, Figure 2, D and E). To investigate the unique functions of these identified MSC subsets, we conducted Gene Ontology (GO) enrichment analysis and assessed their ECM scores. The results showed that FAP^+^ fibroblasts were predominantly involved in ECM and structural organization (Figure 2F and Supplemental Figure 3A), whereas FGFR2^+^ fibroblasts played an important role in the regulation of the inflammatory response (Figure 2G). Moreover, FAP^+^ fibroblasts exhibited the highest ECM-producing capacity of all MSC subsets in the fibrotic state (Figure 2H and Supplemental Figure 3B). The mRNA levels of *FAP* and collagen-related genes were also upregulated in fibrotic areas (Supplemental Figure 3C). Overall, the gene expression enrichment and potential ECM-producing activity of FAP^+^ fibroblasts in fibrotic states indicate that they may play a critical role in driving intestinal fibrosis.

**Figure 3:**
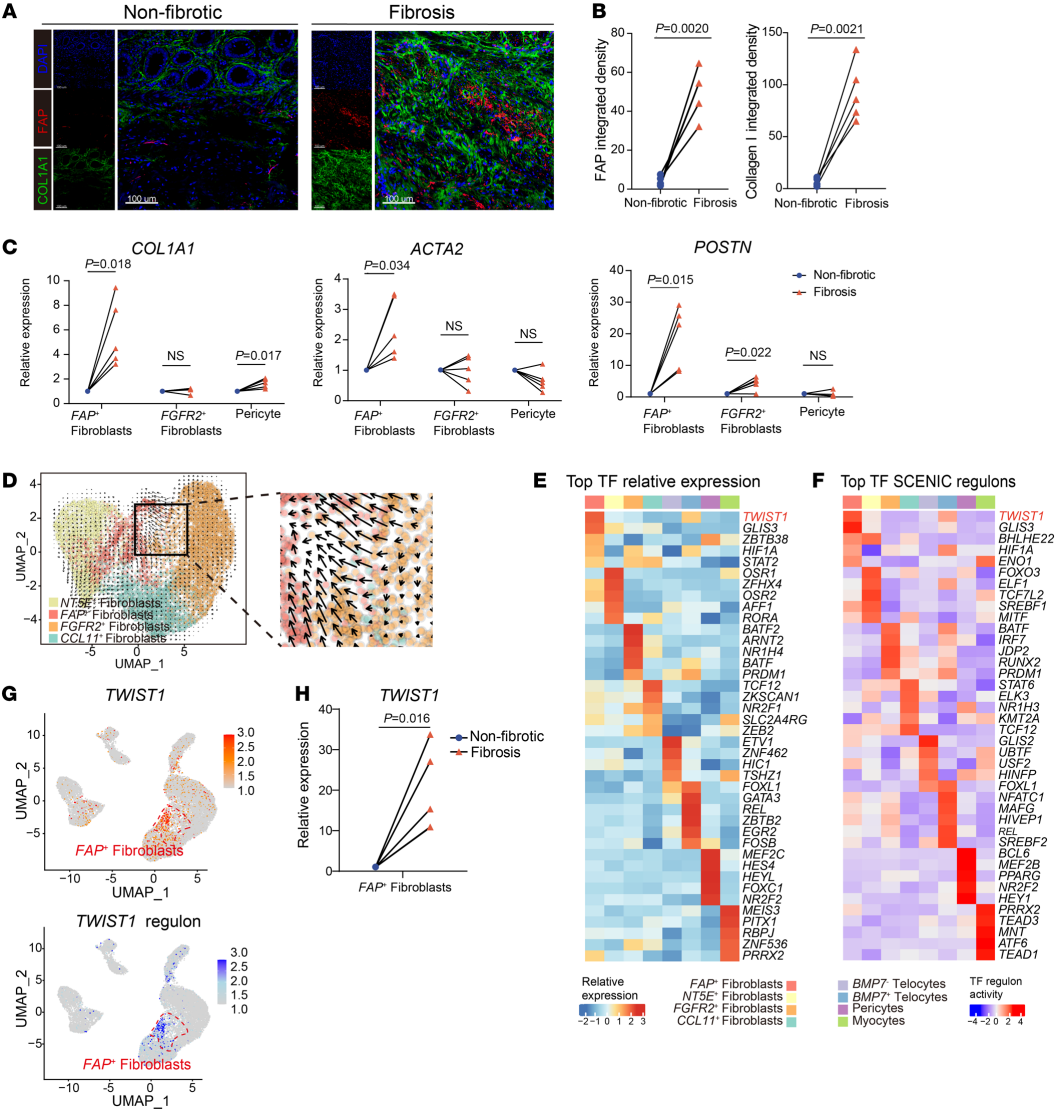
TWIST1 is a critical transcription factor in the differentiation of *FAP*^+^ fibroblasts. (**A**) Representative IF staining of human fibrotic and nonfibrotic intestinal tissue (original magnification, ×20). DAPI (blue), FAP (red), and COL1A1 (green) in individual and merged channels are shown. Scale bar: 100 μm. (**B**) Quantitative analysis (integrated fluorescence intensity) of FAP and COL1A1 in IF staining. The points corresponding to the paired samples (*n* = 5) in the graph are connected. Statistical differences were determined by paired *t* tests. (**C**) The mRNA levels of *COL1A1*, *ACAT2*, and *POSTN* in FAP^+^ fibroblasts, FGFR2^+^ fibroblasts, and pericytes sorted from fibrotic and nonfibrotic sites were analyzed by qPCR. The points corresponding to the paired samples (*n* = 5) in the graph are connected. Statistical differences were determined by paired *t* tests. (**D**) RNA velocity of 4 fibroblast subclusters. Color is as in Figure 2A. The inferred developmental trajectory of FAP^+^ fibroblasts enlarged. (**E**) Heatmap showing the relative expression (*Z* score) of the top 5 transcription factor (TF) genes in each MSC subtype. Color is as in Figure 2A. (**F**) Heatmap showing the normalized activity of the top 5 TF regulons in MSC subtypes predicted by SCENIC. Color is as in Figure 2A. (**G**) Feature plots showing the expression of *TWIST1* (top) and the activity of TWIST1 regulon (bottom). The position of FAP^+^ fibroblasts is red circled. (**H**) The mRNA levels of *TWIST1* in FAP^+^ fibroblasts sorted from fibrotic and nonfibrotic sites were analyzed by qPCR. The points corresponding to the paired samples (*n* = 5) in the graph are connected. Statistical differences were determined by paired *t* tests.

To validate the contributions of FAP^+^ fibroblasts to ECM deposition in the fibrotic areas, immunofluorescence was performed using the intestinal samples from patients with CD. As shown in Figure 3, A and B, FAP^+^ fibroblasts were significantly enriched in the ECM-deposited areas (collagen I) but not in the nonfibrotic intestinal samples. Using FACS and quantitative reverse transcription (qPCR), we examined the mRNA levels of ECM-related genes in FAP^+^ fibroblasts isolated from both fibrotic and nonfibrotic intestinal samples. Our findings further validated the findings of scRNA-Seq analysis and identified the significant upregulation of *COL1A1* (*P =* 0.018), *ACTA2* (*P =* 0.034), and *POSTN* (*P =* 0.015) expression in FAP^+^ fibroblasts within fibrotic areas (Figure 3C and Supplemental Figure 4A). All these findings demonstrate FAP^+^ fibroblasts to be a key pathogenic subset of cells contributing to excessive ECM production in intestinal fibrosis.

To further investigate the origins of the expanded FAP^+^ fibroblast populations in the fibrotic intestine samples, pseudotime analysis of fibroblasts was conducted. RNA velocity and Monocle analysis were utilized to infer the differentiation trajectories. Results indicated that FAP^+^ fibroblasts were originated from FGFR2^+^ fibroblasts (Figure 3D and Supplemental Figure 4, B and C). Upon differentiation into FAP^+^ fibroblasts, we observed the upregulation of several fibrosis-related genes (Supplemental Figure 4D).

### TWIST1 is a critical transcription factor in the differentiation of FAP^+^ fibroblasts.

Since FAP^+^ fibroblasts are differentiated from preexisting fibroblast population, we sought to identify the key transcriptional regulator orchestrating this process and the formation of FAP^+^ fibroblasts. Utilizing single-cell regulatory network inference and clustering (SCENIC) analysis, we found that *TWIST1* exhibited the highest expression level and regulatory activity in FAP^+^ fibroblasts (Figure 3, E–G). qPCR further revealed upregulation of *TWIST1* mRNA levels in fibrotic tissues and FAP^+^ fibroblasts (Figure 3H and Supplemental Figure 4E). Therefore, we proposed that, within the context of CD-affected intestinal tissue, TWIST1 may serve as a key transcription factor (TF) in the differentiation of FAP^+^ fibroblasts.

### Identification of profibrotic macrophage phenotypes and their interactions with FAP^+^ fibroblasts in intestinal fibrosis.

**Figure 4:**
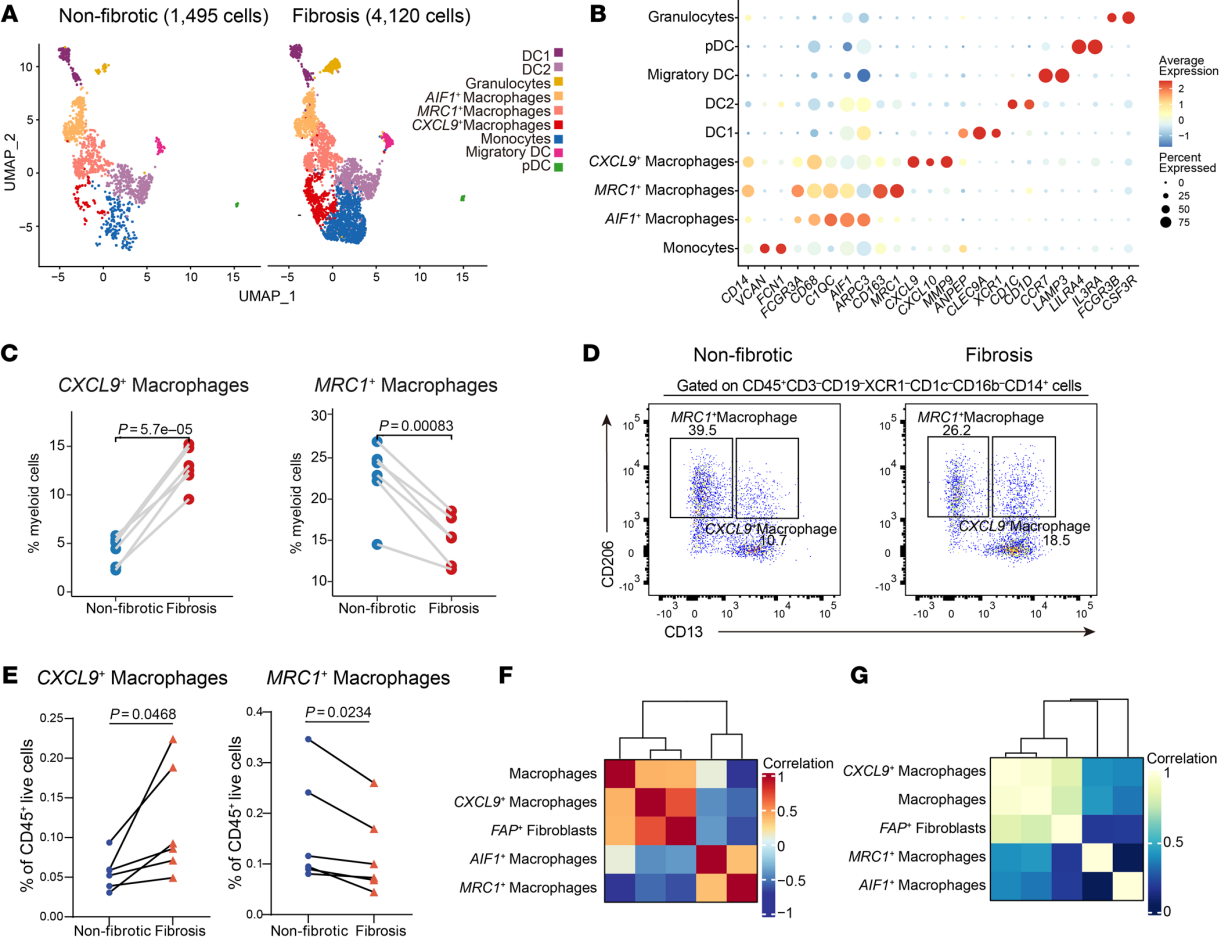
Identification of profibrotic macrophage phenotypes in intestinal fibrosis. (**A**) UMAP plots of the subclustered myeloid cells in the nonfibrotic and fibrotic states. (**B**) Dot plots of the representative markers of subclustered myeloid cells. The average gene expression levels and percentage of cells expressed are shown by dot color and size, respectively. (**C**) Comparison of frequencies of CXCL9^+^ macrophages and MRC1^+^ macrophages of myeloid cells in paired fibrotic intestinal samples (*n* = 6) and nonfibrotic intestinal samples (*n* = 6). Statistical differences were determined by paired *t* tests. (**D**) Representative flow cytometry plots of CXCL9^+^ macrophages and MRC1^+^ macrophages in fibrotic and nonfibrotic mucosa samples. The gating strategies for MSCs are shown in Supplemental Figure 5F. (**E**) Flow cytometry analysis revealed the proportion variation in CXCL9^+^ macrophages and MRC1^+^ macrophages to CD45^+^ live cells in fibrotic and nonfibrotic sites. The points corresponding to the paired samples (*n* = 6) in the graph are connected. Statistical differences were determined by paired *t* tests. (**F**) Heatmap showing the correlation between the percentages of total macrophages and macrophage subsets and FAP^+^ fibroblasts across 12 scRNA-Seq samples. (**G**) Heatmap showing the gene signature correlation between total macrophages and macrophage subsets and FAP^+^ fibroblasts in an RNA-Seq dataset (GSE192786, *n* = 40).

To further understand the microenvironmental trigger of TWIST1 induction in fibroblasts, we further analyzed the heterogeneity of macrophages, the interacting partner of fibroblasts by in-depth clustering. Of the total 5,615 cells retained, which comprised 4,120 cells from fibrotic areas and 1,495 cells from nonfibrotic control areas, we identified 9 distinct clusters based on their marker genes (Figure 4, A and B, and Supplemental Figure 5, A and B). We identified 3 distinct clusters of macrophages: CXCL9^+^ macrophages, MRC1^+^ macrophages, and AIF1^+^ macrophages. Notably, CXCL9^+^ macrophages showed a significant enrichment in fibrotic areas (*P* = 0.000057), whereas the frequency of the other two macrophage clusters diminished in fibrotic areas (Figure 4C and Supplemental Figure 5, C and D). To validate our observations, flow cytometry analysis was performed. We first utilized the single-cell transcriptomic datasets to identify the markers for each identified subtype of the cells (Figure 4B). In the fibrotic intestine, CXCL9^+^ macrophages were significantly increased compared with those in nonfibrotic sites (*P* = 0.0468), while the proportions of MRC1^+^ macrophages and AIF1^+^ macrophages were decreased (Figure 4, D and E, and Supplemental Figure 5, E and F). GO enrichment analysis revealed that CXCL9^+^ macrophages were involved in chemotaxis and extracellular structure organization (Supplemental Figure 6A). We further assessed the roles of these macrophage subsets in fibrosis using classic fibrotic signature scores. As depicted in Supplemental Figure 6, B and C, MRC1^+^ macrophages displayed the highest antifibrotic score, while CXCL9^+^ macrophages displayed the highest profibrotic score, in line with their proportional changes in fibrosis.

**Figure 5:**
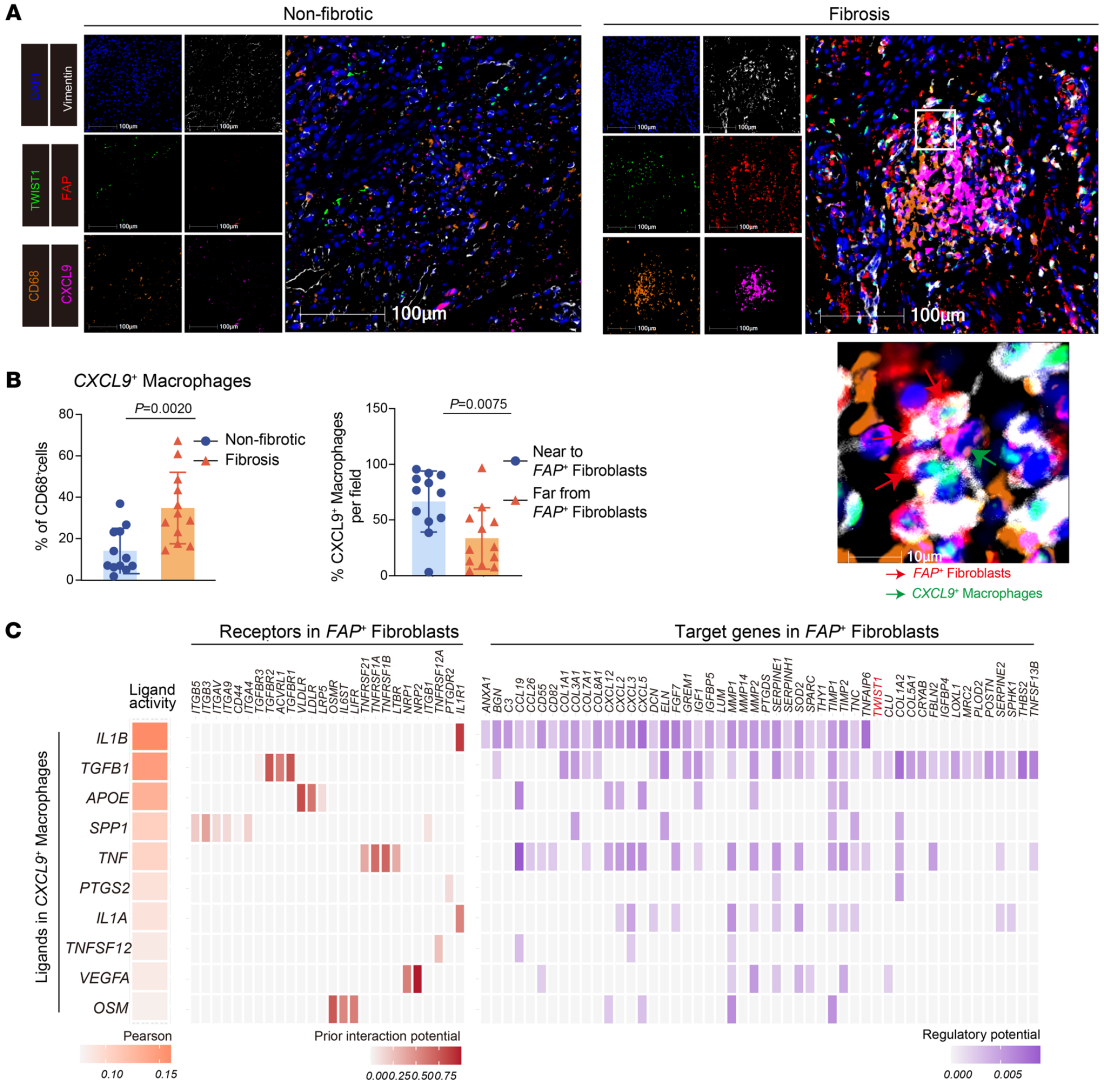
The interaction between *FAP*^+^ fibroblasts and *CXCL9*^+^ macrophages. (**A**) Representative multiplex immunofluorescence (mIF) staining of human fibrotic (right) and nonfibrotic (left) intestinal tissue (original magnification, ×20). DAPI (blue), FAP (red), TWIST1 (green), vimentin (white), CD68 (orange), and CXCL9 (purple) in individual and merged channels are shown. Scale bar: 100 μm. A high-power field (bottom) showing close colocalization between FAP^+^ fibroblasts (red arrows) and CXCL9^+^ macrophages (green arrows). The experiment was performed in 4 patients. (**B**) Quantitative analysis of mIF staining. Proportion of CXCL9^+^ macrophages to CD68^+^ cells between fibrotic and nonfibrotic intestinal samples (left); the proportion of CXCL9^+^ macrophages near to FAP^+^ fibroblasts (within 30 μm) and far from FAP^+^ fibroblasts (of 30 μm) per field in fibrosis states (right) was calculated by HALO software (*n* = 12, 4 patients with 3 fields). Statistical differences were determined by *t* test. (**C**) Heatmap showing the activity of the top-ranked ligands inferred to regulate FAP^+^ fibroblasts by CXCL9^+^ macrophages according to NicheNet (left), the ligand–receptor interaction between them ordered by ligand activity (middle), and the downstream target genes in FAP^+^ fibroblasts (right).

Considering the enrichment of CXCL9^+^ macrophages and FAP^+^ fibroblasts in the fibrotic sites, we sought to investigate their co-occurence in fibrosis development. Pearson’s correlation analysis revealed a markedly positive correlation between the percentages of CXCL9^+^ macrophages and FAP^+^ fibroblasts in the 12 samples (Figure 4F). To validate this finding, we reanalyzed a previously published bulk RNA dataset from intestinal biopsies of patients with CD (GSE192786) to examine the signature correlation between the 2 subsets. As expected, CXCL9^+^ macrophages exhibited a positive correlation with FAP^+^ fibroblasts (Figure 4G). Multiplex immunofluorescence staining also revealed the enrichment of CXCL9^+^ macrophages and close adjacency between these cells and FAP^+^ fibroblasts in the fibrotic site (Figure 5, A and B), indicating the potential interaction between the 2 cell subsets. To further identify the molecular mediators of such interaction, we utilized NicheNet analysis to investigate their interaction patterns. Results indicated that CXCL9^+^ macrophages demonstrated high *IL1B* and *TGFB1* ligand activity, which bound to receptors encoded by *IL1R1*, *TGFBR1*, *TGFBR2*, and *ACVRL1* on FAP^+^ fibroblasts, resulting in the expression of collagen-related genes (Figure 5C). Notably, TWIST1 was predicted to be one of the target genes of the ligand *TGFB1* derived from CXCL9^+^ macrophages, suggesting that CXCL9^+^ macrophages may be involved in the activation of FAP^+^ fibroblasts by upregulating TWIST1. Furthermore, previous studies demonstrated that TWIST1 might be regulated by hypoxia (18–20). GSEA showed more enriched hypoxia pathways in CXCL9^+^ macrophages compared with MRC1^+^ macrophages and AIF1^+^ macrophages (Supplemental Figure 6D), which indicated that the hypoxic niche may be involved in regulating the expression of TWIST1 in FAP^+^ fibroblasts. In conclusion, we identified that profibrotic CXCL9^+^ macrophages were tightly associated with the activation of FAP^+^ fibroblasts and potentially induced the expression of TWIST1 through the IL-1β and TGF-β pathways.

### Transcriptomic homology between murine and human cell subsets.

**Figure 6:**
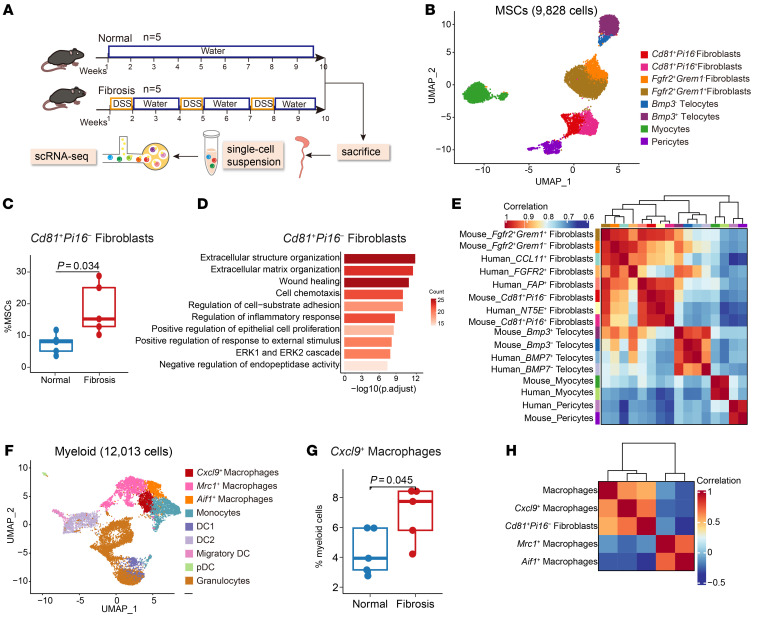
Transcriptomic homology between murine stromal cell subsets and human stromal cell subsets. (**A**) Graphic overview of the scRNA-Seq design for the mouse model. Colons of chronic DSS-treated (*n* = 5) and control mice (*n* = 5) were processed into single-cell suspensions and subjected to scRNA-Seq using 10x Genomics. (**B**) UMAP plot of the subclustered MSCs of the mouse model. (**C**) Box plots showing the proportions of CD81^+^Pi16^–^ fibroblasts in DSS-treated (*n* = 5) and control mice (*n* = 5). Statistical differences were determined by *t* tests. (**D**) Representative Gene Ontology (GO) enrichment of the marker genes expressed in CD81^+^Pi16^–^ fibroblasts. A hypergeometric test was performed with FDR-adjusted *P* values. (**E**) Heatmap showing Spearman’s correlation of transcriptomic homology among human and mouse MSC subclusters. (**F**) UMAP plot of the subclustered myeloid cells of the mouse model. (**G**) Box plots showing the proportions of Cxcl9^+^ macrophages in DSS-treated (*n* = 5) and control mice (*n* = 5). Statistical differences were determined by *t* tests. (**H**) Heatmap showing the correlation between the percentages of total macrophages and macrophage subsets and CD81^+^Pi16^–^ fibroblasts across 10 mouse scRNA-Seq samples.

Since the interaction between FAP^+^ fibroblasts and CXCL9^+^ macrophages seems to be the key feature of the intestinal fibrosis progression in patients with CD, we sought to identify whether similar subsets with comparable functions exist in the chronic dextran sulfate sodium salt–induced (DSS-induced) mouse model of intestinal fibrosis. We isolated cells from the colons of mice treated with water or DSS, respectively, and performed single-cell transcriptome sequencing (Figure 6A). We identified 8 clusters based on their marker genes (Supplemental Figure 7, A and B). Then, we selected MSCs for a secondary round of clustering. We identified 8 subsets at a higher resolution, including 4 fibroblast subsets (Figure 6B and Supplemental Figure 7C). Among these subsets, the abundance of CD81^+^Pi16^–^ fibroblasts was significantly increased in the mouse fibrotic colon (*P* = 0.034, Figure 6C and Supplemental Figure 7D). GO enrichment analysis indicated their involvement in ECM organization (Figure 6D). Consequently, we conducted a comparative analysis of the transcriptional profiles between human and mouse intestinal MSCs. As expected, gene correlation analysis between human and murine fibroblasts showed a degree of cross-species conservation between mouse CD81^+^Pi16^–^ fibroblasts and human FAP^+^ fibroblasts (Figure 6E). Notably, CD81^+^Pi16^–^ fibroblasts also exhibited moderate *Twist1* expression (Supplemental Figure 7E).

To further assess whether the microenvironmental regulation of stromal cells is also evolutionarily conserved, we clustered the mouse myeloid cells into 9 subtypes containing 3 macrophage subsets (Figure 6F and Supplemental Figure 8A). Corresponding to the human data, Cxcl9^+^ macrophages were enriched in the fibrosis model (*P* = 0.045, Figure 6G and Supplemental Figure 8B). GO enrichment analysis of Cxcl9^+^ macrophages revealed their involvement in the immune response and ECM organization (Supplemental Figure 8C). Similar to their corresponding human subsets, Pearson’s correlation analysis showed a markedly positive percentage correlation between Cxcl9^+^ macrophages and CD81^+^Pi16^–^ fibroblasts. (Figure 6H). Overall, we identified murine CD81^+^Pi16^–^ fibroblasts and Cxcl9^+^ macrophages that exhibited transcriptomic similarities to human FAP^+^ fibroblasts and CXCL9^+^ macrophages, respectively, which participated in ECM production and remodeling during fibrosis.

### Targeting TWIST1 inhibits fibroblast activation and attenuates intestinal fibrosis.

**Figure 7:**
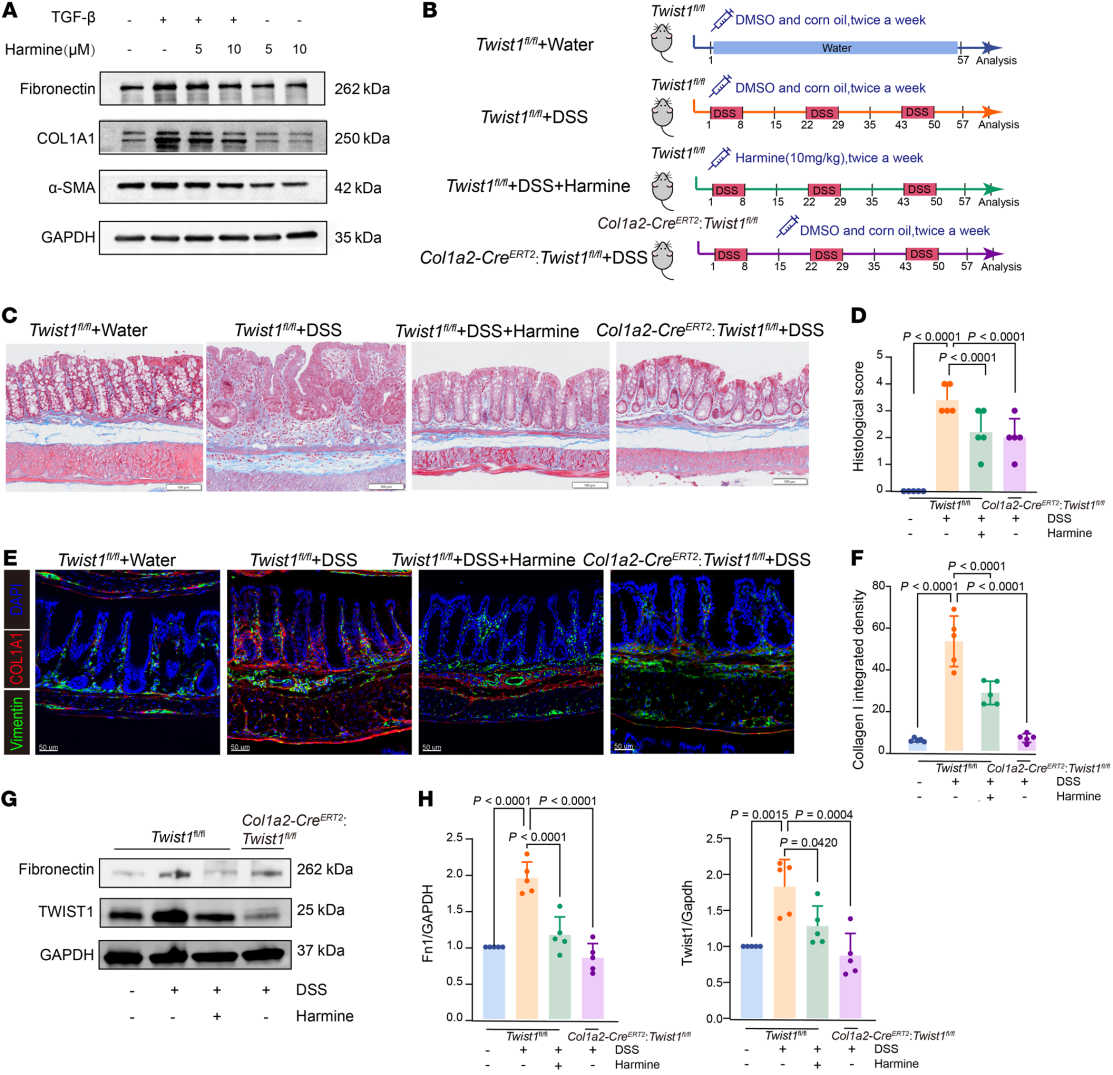
Targeting TWIST1 inhibits fibroblast activation and attenuates experimental intestinal fibrosis. (**A**) Western blotting images showing the expression of ECM-related genes in primary human intestinal fibroblasts with or without TGF-β (5 ng/mL, 48 hours) and harmine administration (5 μM or 10 μM, 48 hours). (**B**) Schematic diagram for the in vivo experiments (5 mice per group). (**C**) Masson’s trichrome staining showing collagen deposition in mouse colons across the 4 indicated groups. Scale bar: 100 μm. (**D**) Bar plots showing histologic scores of mouse colons across the 4 indicated groups. Data represent the mean ± SD. Statistical differences were determined by the 1-way ANOVA with Bonferroni’s correction. (**E**) Representative IF staining of mouse colons across the 4 indicated groups (original magnification, ×20). DAPI (blue), COL1A1 (red), and vimentin (green) in merged channels are shown. Scale bar: 50 μm. (**F**) Quantitative analysis (integrated fluorescence intensity) of COL1A1 in IF staining of mouse colons. Data represent the mean ± SD. Statistical differences were determined by 1-way ANOVA with Bonferroni’s correction. (**G** and **H**) Representative plots (**G**) and quantitative analysis (**H**) of Western blotting images showing the expression of fibronectin and TWIST1 in mouse colons across the 4 indicated groups. Data represent the mean ± SD. Statistical differences were determined by 1-way ANOVA with Bonferroni’s correction.

We identified TWIST1 as a key TFs in the activation of human fibroblasts, which is critical for the process of intestinal fibrosis. To further investigate the function of TWIST1 in regulating fibroblast activation, we treated primary human intestinal fibroblasts with TGF-β, a common stimulator of fibroblast activation. Cells were harvested after 48 hours of stimulation for gene expression tests. TGF-β–treated fibroblasts displayed markedly elevated expression levels of fibronectin, α-SMA, and COL1A1, indicating their transition into an activated state with enhanced ECM production (Figure 7A). We used harmine, a TWIST1 inhibitor that induces its degradation (21), to treat activated fibroblasts. The addition of harmine markedly suppressed the upregulation of fibronectin, α-SMA, and COL1A1 induced by TGF-β (Figure 7A). These results suggest that the inhibition of TWIST1 in vitro could effectively suppress fibroblast activation and ECM production.

To further investigate the in vivo function of TWIST1 in promoting fibroblast activation, we conducted in vivo experiments with a transgenic *Col1a2-Cre^ERT2^* *Twist1^fl/fl^* mouse model. *Twist1* was knocked out particularly in fibroblasts after *Col1a2-Cre^ERT2^* *Twist1^fl/fl^* mice were treated intraperitoneally with tamoxifen daily for 4 days (100 mg per kg body weight each time) at 8 weeks old. A DSS-colitis–induced fibrosis model was established on the mice for further experiments (Figure 7B and Supplemental Figure 9, A and B). The *Col1a2-Cre^ERT2^ Twist1^fl/fl^* group and harmine-treated *Twist1^fl/fl^* group exhibited resistance to DSS-induced weight loss (Supplemental Figure 9C). Masson’s trichrome staining showed that both *Twist1* conditional knockout and pharmacological inhibition led to reduced collagen deposition and histological scores (Figure 7, C and D). To further elucidate the effects of TWIST1 expression on fibroblast activation and ECM production, immunofluorescence staining was conducted. In line with the human data, the expression of TWIST1 and COL1A1 increased in DSS-induced intestinal fibrosis, and targeting TWIST1 effectively inhibited the expression of these genes (Figure 7, E and F, and Supplemental Figure 9D). The expression of ECM-related genes (TIMP-1, COL1A1, COL3A1 and fibronectin) in murine colon tissues was also inhibited by TWIST1 suppression (Figure 7, G and H, and Supplemental Figure 9E). Flow cytometry also demonstrated that after chronic DSS treatment, gp38^+^CD81^+^ MSCs and CD206^–^ macrophages (similar to human FAP^+^ fibroblasts and CXCL9^+^ macrophages, respectively, according to Figure 6E) were significantly reduced in *Col1a2-Cre^ERT2^ Twist1^fl/fl^* mice, but this reduction was not observed under homeostatic conditions (Supplemental Figure 10, A–D). This suggests that knocking out *Twist1* under chronic DSS treatment does indeed affect the abundance of key cell subsets similar to human samples. Collectively, these results indicate that the inhibition of TWIST1 could suppress the activation of fibroblasts and thus attenuate intestinal fibrosis, suggesting its potential therapeutic effect.

## Discussion

Intestinal fibrosis is one of the major complications of typical refractory CD (22). Although multiple studies have elucidated the potential regulatory mechanisms of fibrosis in other organs, such as the lung, kidney, and heart (15, 16, 23), intestinal fibrosis is more complicated owing to its complex cellular composition encompassing almost all known cell types, including epithelial cells, mesenchymal cells, endothelial cells, myeloid cells, lymphocytes, and neuronal and glial cells, and their highly dynamic crosstalk with one another (24). Previous studies have exploited single-cell transcriptomics and revealed the landscape of dysregulated mucosal immunity during CD inflammation with endoscopic biopsies (11, 25, 26). Nevertheless, endoscopic biopsy has only enabled the retrieval of the mucosal layer, overlooking the lesion of submucosal layer resulting from the long-term chronic inflammation of CD (6). To overcome this limitation, we utilized scRNA-Seq on surgical samples from patients with CD with intestinal fibrosis to obtain intact mucosa and submucosa, which are the critical sites of fibrotic pathogenesis. Furthermore, we enriched stromal and hematopoietic cells during the preparation of single-cell suspensions, focusing on the crucial cell-cell interaction driving intestinal fibrosis. Through integrative analysis of these samples, we elucidated the heterogeneity and transcriptomic features of stromal cells in CD intestinal fibrosis, highlighting FAP^+^ fibroblasts as the key cell subset responsible for excessive ECM deposition in fibrosis. These data also illustrated that TWIST1 was a key driver of fibrotic CD and a promising therapeutic target.

Inflammatory bowel disease–related (IBD-related) intestinal fibrosis can be caused by CD or ulcerative colitis (UC). Although the incidence of intestinal stricture is lower in patients with UC compared with that in patients with CD, there is also excessive ECM deposition in the submucosa of inflammatory area in UC (27, 28). Profibrotic cytokine production and ECM remodeling are also presented in fibrotic area of UC, which is similar to CD (28, 29). However, the difference between the two cannot be overlooked either. A study reported an expanded fibroblast subpopulation with expression of fibrosis-related genes, including *FAP* and *TWIST1*, in colons from patients with UC. But the higher expression of *IL11, IL24*, and *IL13RA2* suggested its gene expression pattern was still different from that of FAP^+^ fibroblasts observed in CD from our study (30). Therefore, it is necessary to collect the samples of patients with UC fibrosis for further research to reveal the similarities and differences between the mechanisms of fibrosis in CD and UC.

Previous studies have identified the enrichment of immunosuppressive FAP^+^ fibroblasts secreting ECM and chemokines in tumors, cardiac fibrosis, and interstitial lung diseases (18, 31–34). In those studies, FAP^+^ mesenchymal stromal cells (MSCs) were considered to be the pathogenic subset, and FAP antibody or FAP^+^ CAR T cell–mediated cell ablation ameliorated cardiac fibrosis in a murine model (35). Notably, gremlin 1 (GREM1), a secreted protein, is one of the marker genes of FAP^+^ fibroblasts in our study (Supplemental Table 3). Recent studies have reported that GREM1 was upregulated in intestinal fibrosis and acted as a ligand for VEGFR2 to activate fibroblasts (36, 37). This indicates that, in addition to ECM production, FAP^+^ fibroblasts may promote fibrosis through additional mechanisms like GREM1 secretion. Besides FAP^+^ fibroblasts, another study pointed out the central role of *WNT5A/CDH11* fibroblasts in promoting IBD fibrosis (38). Of note, previous studies have suggested that *WNT5A* fibroblasts are mainly located in the tips of villi that are continuously faced with inflammatory stimuli such as dead cells and invading microbes (39), while intestinal fibrosis is a pathological manifestation that involves the entire layer of the intestine (40). Thus, targeting certain types of fibroblasts might not be sufficient to achieve full recovery from fibrosis, and antifibrotic therapy requires the identification of a more general regulatory program that promotes ECM production in multiple different subsets of stromal cells. In addition, although FAP has long been utilized as a diagnostic and therapeutic target for fibrosis-related diseases (41), the FAP protein itself functions as a serine protease and primarily participates in the remodeling of ECM substrates such as collagen degradation (42–44), and direct inhibition of FAP^+^ cells may not be an ideal strategy to attenuate intestinal fibrosis. Although a previous study showed that anti-FAP treatment could reduce type I collagen and TIMP-1 production by CD strictures, the efficacy and safety of this therapy have not been confirmed in vivo (45).

In our study, we also identified the close interaction between CXCL9^+^ macrophages and FAP^+^ fibroblasts in the pathogenesis of intestinal fibrosis via IL-1, TGF-β, and OSM production. Distinct macrophage subtypes could be involved in fibrogenesis, which may open up new therapeutic perspectives in the treatment of intestinal fibrosis (46). A previous study found that M2 macrophages could stimulate the proliferation of MSCs upon hypoxia through TGF-β production in the TNBS rat model (47). Therefore, precision targeting of macrophage subsets and pathogenic molecules is essential for the treatment of intestinal fibrosis.

Our study revealed that TWIST1 was a key TF driving ECM production in FAP^+^ fibroblasts and that the inhibition of TWIST1 significantly suppressed fibroblast activation and attenuated intestinal fibrosis. TWIST1 is a member of the TWIST proteins, which belong to the large family of basic helix-loop-helix TFs (48). It promotes the epithelial-mesenchymal transition in cancers, granting oncogenic and metastatic properties to tumors (49, 50). Lovisa et al. reported that TWIST1 facilitated the endothelial-mesenchymal transition in kidney tissue, which contributes to fibrosis (51). Moreover, TWIST1 has been demonstrated as a TF that drives fibroblast activity and ECM production (52). Our findings agree with the results of these studies. However, the factors that trigger the upregulation of TWIST1 in FAP^+^ fibroblasts remain unclear. Hypoxia in the tumor and fibrosis microenvironment may induce the expression of TWIST1 (18–20). As an inhibitor of TWIST1, harmine can inhibit the expression and result in the degradation of TWIST1; it was identified as a first-in-class TWIST1 inhibitor with marked antitumor activity in oncogene-driven non–small cell lung cancer (53). In addition, harmine was reported to inhibit renal fibrosis through regulation of lipid metabolism and to suppress the fibrogenesis of fibroblasts in keloid, suggesting its antifibrotic potential (54, 55). Consistently, our study showed that harmine administration or TWIST1 deletion relieved ECM deposition in mice exposed to chronic DSS-induced fibrosis. These findings suggest that TWIST1 inhibition is indeed a promising strategy for IBD fibrosis treatment. Although harmine has a significant inhibitory effect on TWIST1, it has many pharmacological activities via associated mechanisms (56). Further study of its wider antifibrosis effects is needed.

The study is subject to limitations due to the relatively limited number of patients included and the lack of individuals acting as healthy controls. In addition, since our samples are all from patients with CD, whether the molecular and genetic characterization of fibrosis tissue presented in this study is suitable for UC fibrosis still needs further study. Finally, the study is further limited by incomplete understanding of the mechanisms of how TWIST1 regulates the activation of FAP^+^ fibroblasts.

In conclusion, we elucidated the heterogeneity of stromal cells in intestinal fibrosis and identified FAP^+^ fibroblasts as the crucial subset driving the fibrosis responsible for excessive ECM deposition. Furthermore, we found that TWIST1 is a critical TF in fibroblast activation and that the inhibition of TWIST1 could attenuate intestinal fibrosis. Our study highlights the potential therapeutic value of targeting TWIST1 in preventing the development and progression of intestinal fibrosis.

## Methods

### Sex as a biological variable.

Our study examined male and female patients and animals, and similar findings are reported for both sexes.

### Human intestinal specimens.

The terminal ileum containing stricture and adjacent nonfibrotic segment from patients with CD who underwent intestinal resection for fibrotic stenosis was collected. Radiology and/or failure to pass an ileocolonoscope were used to determine the presence of intestinal fibrosis prior to resection. Following resection, fibrosis and nonfibrotic tissues were identified based on gross anatomy. Fibrosis (with the presence of stricture) and nonfibrotic tissues (distal to the stricture without the presence of stricture and inflammation) were obtained from the same patient’s resection. A experienced IBD pathologist assessed and classified each tissue based on a histopathologic fibrosis score (57). Demographics and clinical information of included patients with CD are displayed in Supplemental Table 1.

### Mice.

The *Col1a2-Cre^ERT2^* mouse line and *Twist1^fl/fl^* mouse line were generated by Shanghai Model Organisms Centre. The *Col1a2-Cre^ERT2^* mice have previously been described (58, 59). Construction strategy and genotype identification of *Twist1^fl/fl^* mice were shown in Supplemental Figure 9, A and B. Genomic DNA was isolated from mouse tail. Tissues were lysed by incubation with proteinase K at 55°C overnight, followed by centrifugation at 9,600*g* for 2 minutes to obtain supernatant with genomic DNA. DNA was precipitated by adding equal volume proportion of isopropanol and was washed in 70% ethanol. Specific primers used for distinguish of the *Twist1^fl/fl^* allele and the wild-type allele are listed in Supplemental Table 6. To generate *Col1a2-Cre^ERT2^ Twist1^fl/fl^* mice, we crossed *Col1a2-Cre^ERT2^* mice with *Twist1^fl/fl^* mice for several generations before subsequent experiments. All mice were bred and maintained at accredited animal facilities under specific pathogen–free conditions in standard cages on a strict 12-hour-day/night cycle at 22°C–24°C and allowed free access to water and a standard diet. Unless otherwise indicated, age- and sex-matched mice were used in all assays.

### Animal model experiments.

*Twist1^fl/fl^* mice and *Col1a2-Cre^ERT2^Twist1^fl/fl^* cohoused littermates (8 weeks old) were injected intraperitoneally with tamoxifen daily for 4 days (100 mg per kg body weight each time). The mice were subjected to 3 cycles of DSS administration (7 days of DSS administration followed by 14 days of regular drinking water) according to previous study (60). Harmine (MedChemExpress, HY-N0737A, 10 mg/kg) was dissolved in 10% DMSO and 90% corn oil and injected intraperitoneally twice a week during each DSS cycle (regularly on Tuesday and Friday weekly). The dosage of harmine was determined based on previous study (54). The use standardization of harmine was determined according to the manufacturer’s instructions (https://www.medchemexpress.cn/harmine.html). In other control groups, the mixture of 10% DMSO and 90% corn oil with the same volume were injected intraperitoneally. All mice were then sacrificed on day 65, and colon tissues were taken for histological analysis, qPCR, and Western blot.

### Histological sections preparation and evaluation.

Fresh intestinal tissues from the patients with CD and mouse models were subsequently fixed in 4% paraformaldehyde for 24 hours, then transitioned to 70% ethanol for another 24 hours, and embedded in paraffin. Formalin-fixed, paraffin-embedded blocks of CD intestinal tissues were then cut into 4 μm serial sections for H&E staining and Masson’s trichrome staining. The pathological score (methods from Adler J, et al., ref. 57) and the thickness of mucosa, submucosa, and muscularis propria layer were evaluated by a specialized IBD pathologist.

### Single-cell suspension processing from mouse colon.

C57BL/6 mice were housed in specific pathogen–free housing at Shanghai Model Organisms Center. For chronic DSS, C57BL/6 mice were subjected to 3 cycles of DSS administration (7 days of DSS administration followed by 14 days of regular drinking water). The mouse colons from chronic DSS and control groups were surgically excised, flushed with PBS, opened longitudinally, and cut into 4 equal pieces by length. The intestinal tissues were then washed with RPMI 1640 containing 10% FBS and cut into pieces of approximately 0.25 cm in length before being digested in RPMI 1640 containing 10% FBS, collagenase type VIII (50 U/mL) and DNase I (50 U/mL) at 37°C for 60 minutes. After digestion, the remaining tissue fragments were collected into a 15 mL tube, vortexed vigorously for 30 seconds, and passed through a 70 μm cell strainer. The resultant cell suspension was then centrifuged at 757*g* and 4°C for 5 minutes before the supernatant was discarded. At this point, freshly prepared cell suspensions were ready for scRNA-Seq.

### Single-cell suspension processing from human intestine.

Freshly resected intestinal tissues from patients with CD were processed. The period between resection in the operating room and beginning tissue processing in the laboratory was less than 30 minutes. Fat tissue and visible blood vessels were removed before subsequent processing. Fresh mucosa and submucosal layers of nonfibrotic and stricture tissue were dissected, washed with ice-cold PBS, and cut into small pieces. Tissues were placed and shaken into EDTA-containing buffer (5 mM EDTA, 15 mM HEPES, 1 mM DTT, and 10% FBS-supplemented PBS) for 45 minutes at 37°C. After that, small tissue pieces were minced and digested with collagenase VIII at 0.38 mg/mL and DNase I at 0.1 mg/mL in DMEM (containing 10% FBS, 100 U/mL penicillin, and 100 mg/mL streptomycin) for 50 minutes at 37°C. After digestion, cells were filtered through a 75 μm filter. Freshly prepared cell suspensions were assessed for viability with Trypan blue and counted. Single-cell suspensions with no or minimal clumps and viability greater than 80% were ready for scRNA-Seq and flow cytometry staining.

### Single-cell RNA-Seq library preparation and sequencing.

Intestinal single cells were resuspended in PBS supplemented with 0.04% BSA. Single-cell transcriptomic amplification and library preparation were performed using 10 X Chromium 3′ v3 kit (10x Genomics) according to manufacturer’s instructions. Sequencing was performed on a NovaSeq 6000 platform in Shanghai Institute of Immunology.

### Single-cell RNA-Seq data processing.

Standard pipelines of Cell Ranger (10x Genomics) were used to do sequence processing, mapped to the reference genome (human, GRCh38; mouse, mm10) using cellranger v5.0.1. Then, the preliminary count matrices generated were analyzed using the R package Seurat v4.1.1 (61). For human intestinal specimens, the matrix was then filtered to remove genes expressed in fewer than 3 cells, cells with fewer than 500 or more than 6,000 genes; and with UMI counts of less than 500 or more than 30,000 and with greater than 20% of mitochondrial genes. For mouse samples, cells with less than 500 UMI counts and 200 detected genes, with UMI counts above 40,000 and detected genes above 6,000 and that contained more than 25% mitochondrial gene counts were filtered out. To remove potential doublets, we used Python package Scrublet v0.2.3 (62) to identify potential doublets with default parameter. The expected doublet rate was set to be 0.08, and cells predicted to be doublets were filtered. After quality control, a total of 91,316 cells from surgical specimens and 83,337 cells from mouse model samples were remained. Then, the count data per cell were normalized and transformed to log scale by “NormalizeData” function in Seurat.

### Dimension reduction and clustering analysis.

Dimension reduction and unsupervised clustering were performed according to the standard workflow in Seurat. We scaled data with the top 2,000 most-variable genes by using “FindVariableFeatures” function in R package Seurat. Subsequently, the expression levels of genes were scaled by regressing out the unwanted sources of variation, including total counts, percentages of mitochondrial gene counts, and percentages of ribosomal gene counts. Then, we used variable genes for principal component analysis, and the top 20 components were used for downstream analyses. To eliminate the batch effect, we performed harmony algorithm in R package Harmony v0.1.0 (63) to remove batch effect before clustering analysis and applied FindNeighbors and FindCluster in Seurat to obtain cell subtypes. Finally, a uniform manifold approximation and projection (UMAP) dimensionality reduction was performed on the Harmony dimensions (RunUMAP function). We used R package Clustree v0.5.1 (64) to find a reasonable resolution parameter for the function “FindClusters” in Seurat. Cells form human intestinal tissues, and mouse model samples were clustered at 2 stages of the analysis separately. After the first round of unsupervised clustering, we annotated major cell types, including T/ILCs cells, B cells, plasma cells, myeloid cells (neutrophils, monocytes, macrophages, DCs, and mast cells), epithelial cells, endothelial cells, MSCs, and glial cells according to canonical known cell markers. For the second step, we performed unsupervised clustering on MSCs and myeloid cells from human and mouse model samples, respectively. In total, a high-resolution map of 24 cell clusters in human intestinal tissue and 23 cell clusters from mouse model were obtained.

### Analysis of differentially expressed genes.

We used the “FindAllMarkers” function in Seurat to identify genes that are differentially expressed between clusters with the following parameters: min.pct = 0.1, logfc.threshold = 0.25, only.pos = T. The nonparametric Wilcoxon’s rank-sum test was used to obtain *P* values for comparisons, and the adjusted *P* values, based on Bonferroni’s correction, for all genes in the dataset. We used heatmap to visualize differentially expressed genes (DEGs) based on gene expression after the log transformation and scaling. A comprehensive list of both canonical and signature marker genes for each cell cluster has been included in Supplemental Tables 3 and 4.

### Functional annotation and GSEA analyses.

We calculated ECM gene signature score using genes of Extracellular Matrix Organization (GO:0030198) from Gene Ontology Resource. The gene sets used to calculate the functional scores across MSCs clusters (collagen score, glycoprotein score, proteoglycan score), myeloid cell clusters (profibrosis score, antifibrosis score) were downloaded from published papers (16, 65) and summarized in Supplemental Table 5. The normalized expression matrix of genes included in 1 gene set were used, and the mean value of all genes in the gene set of each cell was calculated as the gene signature score of the cell.

We used enrichGO function in R package clusterProfiler v4.2.2 (66, 67) to identify the significantly differential enrichment of GO biological process gene sets. We also downloaded 50 hallmark gene sets from the Molecular Signatures Database (MSigDB, http://software.broadinstitute.org/gsea/msigdb/) and used GSEA function in R package clusterProfiler v4.2.2 to identify the significantly differential enrichment of annotated gene sets between CXCL9^+^ macrophages and the other 2 macrophage clusters. We considered gene signatures or pathways with FDR < 0.05 as significantly enriched.

### Trajectory inference analyses.

To investigate the origin of differentiation for FAP^+^ fibroblasts, we analyzed expression dynamics by estimating gene splicing and degradation rates using explicit measurements of newly transcribed pre-mRNA (unspliced) and mature mRNA (spliced). We used the R package velocyto.R v0.6 (68) to calculate the RNA velocity value of each gene in each cell and embed the RNA velocity vector in a low-dimensional space and then visualized it on the UMAP projection. To verify the differentiation results inferred by velocyto.R, we also used R package Monocle2 v2.14.0 (69) to conduct pseudotime transitional trajectory of 4 fibroblast subsets. The top 2,000 highly variable genes in fibroblasts were selected as input, and dimensionality reduction was performed by “DDRTree” method. DEGs along the pseudotime trajectory were identified by the ‘‘differentialGeneTest’’ function with a *q* value of less than 0.01 and visualized by “plot_pseudotime_heatmap” function.

### TF regulon analysis.

The analysis of the regulatory network and regulon activity was performed by R package SCENIC v1.1.3 (70). The regulon activity (measured in AUC) was analyzed by AUCell module of the SCENIC, and the active regulons were determined by AUCell default threshold. The differential-expression regulon was identified by Wilcoxon’s rank-sum test in “FindAllMarkers” function in R package Seurat with following parameters: min.pct = 0.05, logfc.threshold = 0.15, pseudocount.use = F, only.pos = T. The scaled expression of regulon activity was used to generate a heatmap.

To check the gene expression of TFs alone, we retrieved genes encoding TFs from 4 TF-related public datasets: JASPAR (71) (http://jaspar.genereg.net/), Transcription factor prediction database (DBD) (72) (https://transcriptionfactor.org/), AnimalTFDB (73) (http://bioinfo.life.hust.edu.cn/AnimalTFDB/), and F2DNA (74) (http://www.fiserlab.org/tf2dna_db/). We overlapped the TF genes with the DEGs quantified above and determined the most specifically expressed TFs in each cluster.

### Cell-cell communication analysis.

We used R package nichenetr v1.0.0 (75) to infer the mechanisms of interaction between CXCL9^+^ macrophages and FAP^+^ fibroblasts. For ligand and receptor interactions, clustered cells with gene expression over 10% were considered. The top 20 ligands and top 500 targets of DEGs of “sender cells” and “affected cells” were extracted for paired ligand-receptor activity analysis. When evaluating the regulatory network of CXCL9^+^ macrophages on FAP^+^ fibroblasts, FAP^+^ fibroblasts was considered as receiver cells and the other 7 MSCs subclusters were used as reference cells to check the regulatory potential of CXCL9^+^ macrophages on FAP^+^ fibroblasts, The ligand_activity_target_heatmap in Nichenet_output was used to show the regulatory activity of ligands. Activity scores ranged from 0 to 1.

### Analysis of public RNA-Seq data.

Expression RNA-Seq dataset of fibrotic signatures in patients with CD was downloaded from GEO (GSE192786, *n* = 40). The signature scores of *total* macrophages and macrophage subsets and FAP^+^ fibroblasts of each sample were calculated by the mean log_10_ normalized expression across all signature genes, according to the marker genes identified in single-cell sequencing (logFC > 0.25 and adjusted *P* < 0.05). Pearson’s correlation analysis was performed to assess the association between the expression of total macrophages and macrophage subsets signature and FAP^+^ fibroblasts signature.

### Correlation analysis of MSCs subsets between human and mouse.

To analyze the transcriptomic homology between human and mouse MSCs subsets, we used the “convert_human_to_mouse_symbols” function in R package NicheNet to convert human gene names in scRNA-Seq data to corresponding mouse gene names, and intersected them with genes from mouse scRNA-Seq data, ultimately retaining 15,071 shared genes. Then, we used top 2,000 most-variable genes for downstream analysis by using “FindVariableFeatures” function in Seurat. Integration between human and mouse MSCs scRNA-Seq data was performed by “FindIntegrationAnchors” and “IntegrateData” function. The mean normalized expression across all variable genes for each annotated MSCs subcluster was calculated. Spearman’s correlation analysis was performed to assess the association between the human and mouse MSCs subsets based on the above “mean expression-MSCs subsets” matrix.

### Flow cytometry.

Freshly prepared single-cell suspensions were washed and incubated with Live/Dead dye (BV510, Biolegend) in PBS at 4°C for 10 minutes. After that, cells were washed in PBS with 2% FBS and 2 mM EDTA (FACS buffer). In order to reduce nonspecific binding of proteins, myeloid cells were stained with 1:50 human Fc block at 4°C for 20 minutes. Subsequently, cells were incubated with antibodies in the dark at 4°C for 30 minutes. Finally, labeled cells were washed twice and resuspended with FACS buffer. Flow cytometry analysis was performed on a BD Symphony (BD Biosciences). BD FACSAria III cell sorter (BD Biosciences) was used to sort live stromal cells. We obtained data by using BD FACSDiva software v8.0.2 and analyzed data with FlowJo v.10.81. For stromal cell subset analysis, the following antibodies were used: anti-CD45 (Biolegend, 368536); anti-CD31 (Biolegend, 303110); anti-CD326 (BD Horizon, 748381); anti-CD146 (Biolegend, 361022); anti-CD142 (eBioscience, 12-1429-41); anti-CD90 (Biolegend, 328142); anti-CD34 (Biolegend, 343514); anti-FAP (RD System, FAB3715A); and anti-CD26 (BD OptiBuild, 745244). Antibodies for myeloid cells included anti-CD45 (BD Horizon, 563792); anti-CD3 (BD Horizon, 563725); anti-CD19 (BioLegend, 302240); anti-CD1c (BioLegend, 331524); anti-XCR1(BioLegend, 372608); anti-CD14 (BioLegend, 301822); anti-CD16b (BD OptiBuild, 744968); anti-CD206 (BD, 564063); and anti-CD13 (BioLegend, 301704).

### Immunofluorescence staining and imaging.

Fresh tissues were fixed in 1% paraformaldehyde at 4°C overnight, dehydrated with 30% sucrose over 12 hours, and transferred to OCT and frozen in –80°C for use. Tissues were sectioned into 10 μm slices and rehydrated in PBS for 10 minutes. Permeabilization was done by soaking slices into precooled methanol for 30 minutes at −20°C. Sections were blocked with blocking buffer (0.3% Triton X-100, 1% BSA, 1% FBS, and 0.1 mol/L Tris-HCL buffer) supplemented with goat serum. The slides were then incubated with primary antibodies at 4°C overnight (3 hours at room temperature for fluorochrome-conjugated primary antibodies) and washed with PBS, followed by incubation with fluorochrome-conjugated secondary antibodies for 1 hour at room temperature. After washing, sections were counterstained for nuclei and mounted with DAPI Fluoromount-G (Southernbiotech, 0100-20) and coated with coverslips. Images were observed with Olympus microscopy and analyzed with Imaris Version 9.0.1. Quantitative analysis was performed by ImageJ (NIH). The following antibodies were used for IF staining: FAP (Novus Biologicals, FAB3715G-100, 1:100); COL1A1 (CST, 72827S, 1:50); Vimentin (CST, 9854S, 1:200); PDPN (Biolegend, 127406, 1:200); TWIST1 (Abcam, ab175430, 1:200); and goat anti-rabbit (Abcam, ab150080, 1:500).

### Multiplex immunofluorescence staining.

Human intestinal tissues from patients were placed in 4% paraformaldehyde for 24 hours, dehydrated, and embedded in paraffin and sectioned into 5 μm slices for use. Sections were stained using PanoPANEL Kits (panovue, 10234100050) to perform multiplex immunofluorescence according to the manufacturer’s instructions. Briefly, slides were deparaffinized with xylene and a graded series of ethanol dilutions (100%, 95%, and 70%), followed by microwave-based antigen retrieval using the antigen restoration solution and antibody blocking for 30 minutes. Primary antibodies were incubated for 1 hour at room temperature, and HRP-labeled secondary antibodies were incubated at room temperature for 30 minutes, followed by TSA fluorescent dye working solution incubation for 30 minutes. Finally, after multiantigen staining, nuclei were stained with DAPI for 20 minutes. Slides were enclosed using nail polish, scanned using the SLIDEVIEW VS200 (Olympus), and analyzed with HALO software. The following antibodies and corresponding fluorescent dyes were used for multiplex immunofluorescence staining: FAP (Abcam, ab218164, 1:100, PPD480); TWIST1 (Abcam, ab175430, 1:200, PPD520); CD68 (Abcam, ab955, 1:200, PPD570); CXCL9 (Abcam, ab290643, 1:100, PPD650); and Vimentin (Abcam, ab8978, 1:200, PPD780).

### Primary human intestinal fibroblasts.

Three to 4 strips of mucosa were mechanically dissected from the intestinal mucosa specimens of patients with CD. First, the strips were incubated in dithiothreitol for 30 minutes, and then they were transferred into Hank’s Balanced Salt Solution along with penicillin and streptomycin for 3 hours. Subsequently, the strips were minced into small pieces (2–3 mm^2^) using a scalpel. These mucosa pieces were then placed onto a prescored 100 mm tissue culture dish and allowed to adhere for about 15 minutes. Afterward, the plate was flooded with Dulbecco’s minimal essential medium supplemented with 10% FBS and antibiotics. The outgrowing cells were cultured to confluence and established as long-term cultures. These cultures were fed twice a week and subcultured at confluence. The fibroblasts were utilized between passage 3 and 10.

### Fibroblast stimulation assay.

Human intestinal fibroblasts were isolated from the intestinal specimens of patients with CD (methods from Zhao S, et al., ref. 76). The primary fibroblasts were cultured in RPMI 1640 medium supplemented with 10% FBS and 100 U/mL penicillin and streptomycin. Subsequently, various combinations of 5 ng/mL TGF-β (RD, 7754-BH/CF) and 5 μM or 10 μM harmine (MedChemExpress, HY-N0737A) were added (Figure 7A). Harmine was dissolved in DMSO according to the manufacturer’s instructions (https://www.medchemexpress.cn/harmine.html). The dosage of harmine was determined based on previous study (77). After incubating 48 hours at 37°C in 5% CO2 incubator, cells in every well were harvested and used for Western blot test.

### Western blot.

The indicated cells were washed with cold PBS twice, collected with a cell scraper, and treated with RIPA lysis buffer (Beyotime, P0013B) on ice for 5 minutes. Prior to homogenization, a protease inhibitor cocktail (Thermo Fisher Scientific, 78442) was added. Then, the whole solution was subjected to centrifugation at 12,000*g* for 10 minutes. The supernatant was collected, and protein loading was normalized with BCA assay. The total protein (20 μg) was then subjected to 10% SDS-PAGE and transferred to PVDF membranes. With incubation of 5% skimmed milk for 1 hour at room temperature, the membranes were incubated with primary antibodies in 4°C overnight. The blots were washed with TBST for 5 minutes (3 times) and exposed for 60 minutes at room temperature to an appropriate HRP- linked secondary antibody (Anti-mouse IgG, HRP-linked Antibody, CST, 7076; Anti-rabbit IgG, HRP-linked Antibody CST, 7074). The detection was achieved using the enhanced chemiluminescence system (Tanon 5200 Mui). The following primary antibodies were used: Fibronectin (Abcam, ab268020, 1:1,000); COL1A1 (CST, 72026S, 1:1,000); α-SMA (MilliporeSigma, A2547, 1:1,000); Twist1 (Abcam, ab175430, 1:1,000); and GAPDH (CST, 2118S, 1:1,000).

### qPCR.

Total RNA was extracted from intestinal tissue or sorted intestinal single-cell suspension using Trizol (Invitrogen). cDNA was then synthesized using the SuperScript III cDNA Synthesis Kit (Invitrogen). mRNA expressions were detected with SYBR Green on a 96 well real-time PCR system (Applied Biosystems, viia7). Primers were obtained from PrimerBank. Primer sequences are listed in Supplemental Table 2. Relative mRNA expression was calculated using the 2^–ΔΔCt^ method.

### Statistics.

Statistical analysis was done by R or GraphPad Prism 6, and *P* < 0.05 was considered as significant. Two-sided, 2-tailed *t* test, Wilcoxon’s rank-sum, 1-way ANOVA, or Kruskal-Wallis test were used and are indicated in figure legends. Bonferroni’s correction was performed for multiple comparison. *P* values of less than 0.05 were considered significant.

### Study approval.

All clinical sample collection procedures were approved by local medical ethnics from Ruijin Hospital Affiliated to Shanghai Jiao Tong University School of Medicine (no. 2020-333). Informed written consent and patient assent were obtained from all included patients before surgery. Patients or the public were not involved in the design, conduct, reporting, or dissemination plans of this research. The animal experiments were approved by Institutional Animal Care and Use Committee of Shanghai Model Organisms Center Inc. (no. 2023-0020) and were carried out following the Institutional Ethical Guidelines for Experiments with Animals, as well as the *Guide for the Care and Use of Laboratory Animals* (National Academies Press, 2011).

### Data availability.

Raw sequencing reads of all single-cell experiments for human samples have been deposited in the Genome Sequence Archive for Human (GSA-Human, https://ngdc.cncb.ac.cn/gsa-human/), with data accession HRA006083 under project PRJCA021346. Raw sequencing reads of single-cell experiments for mouse samples have been deposited in GSA (https://ngdc.cncb.ac.cn/gsa/), with data accession CRA016292 under project PRJCA021346. The processed public bulk RNA-Seq dataset of patients with CD with intestinal fibrosis (GSE192786) was downloaded from Gene Expression Omnibus (GEO; https://www.ncbi.nlm.nih.gov/geo/). Values for all data points in graphs are reported in the Supporting Data Values file.

## Author contributions

BS, DZ, and NW conceived and designed the research and supervised the studies. JW and ZX conducted analyses. Y Zhang, ZH, YG, LZ, and CZ conducted the clinical cohort and collected samples. Y Zhang, JW, Y Zhao, SS, QY, and JQ performed the experiments. YY, HS, and NW were involved in interpretation. Y Zhang and HS wrote the manuscript. DZ and BS revised the manuscript. All authors read and approved the final manuscript. The order of the co–first authors’ names was assigned on the basis of the academic contribution of each author.

## Supplementary Material

Supplemental data

Unedited blot and gel images

Supporting data values

## Version 1 07/18/2024

In-Press Preview

## Version 2 09/17/2024

Electronic publication
