# Supplementary material for: TWIST1+FAP+ fibroblasts in the pathogenesis of intestinal fibrosis in Crohn’s disease
Source: J Clin Invest. 2024 Jul 18;134(18):e179472. doi: 10.1172/JCI179472 (PMC11405050; doi:10.1172/JCI179472)
Supplement: Supplemental data [file jci-134-179472-s182.pdf]

Supplemental figures

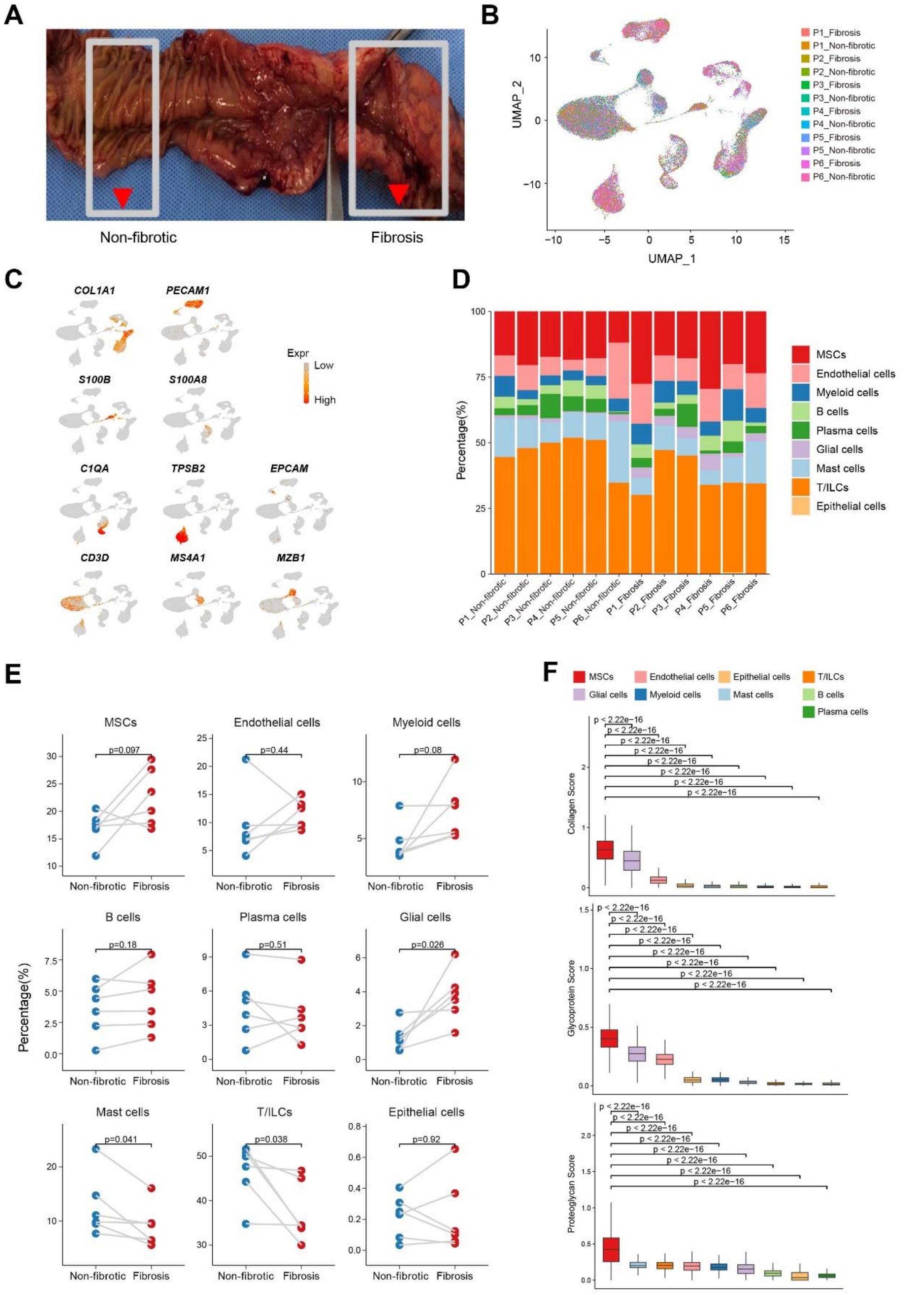

Supplemental Figure 1. Single-cell sequencing reveals cellular landscape of intestinal fibrosis.

(**A**) Representative gross pathology images of non-fibrotic and fibrotic site of surgical intestinal specimens from a CD patient.

(**B**) UMAP plot showing all cells distribution of across 6 fibrotic and 6 non-fibrotic

samples. R package harmony was used to correct batch effects and constructed one UMAP based on all cells.

(**C**) Feature plots showing the expression of canonical markers in major cell types from CD patients.

(**D**) Bar plots showing percentage of major cell types across 12 samples.

(**E**) Comparison of frequencies of major cell types in paired fibrotic intestinal samples (n = 6) and non-fibrotic intestinal samples (n = 6). Statistical difference were determined by paired t tests.

(**F**) Boxplots showing respective ECM signature score (collagen, glycoprotein and proteoglycan) of each cell type in fibrosis states. Statistical differences were determined by one-way ANOVA with Bonferroni correction.

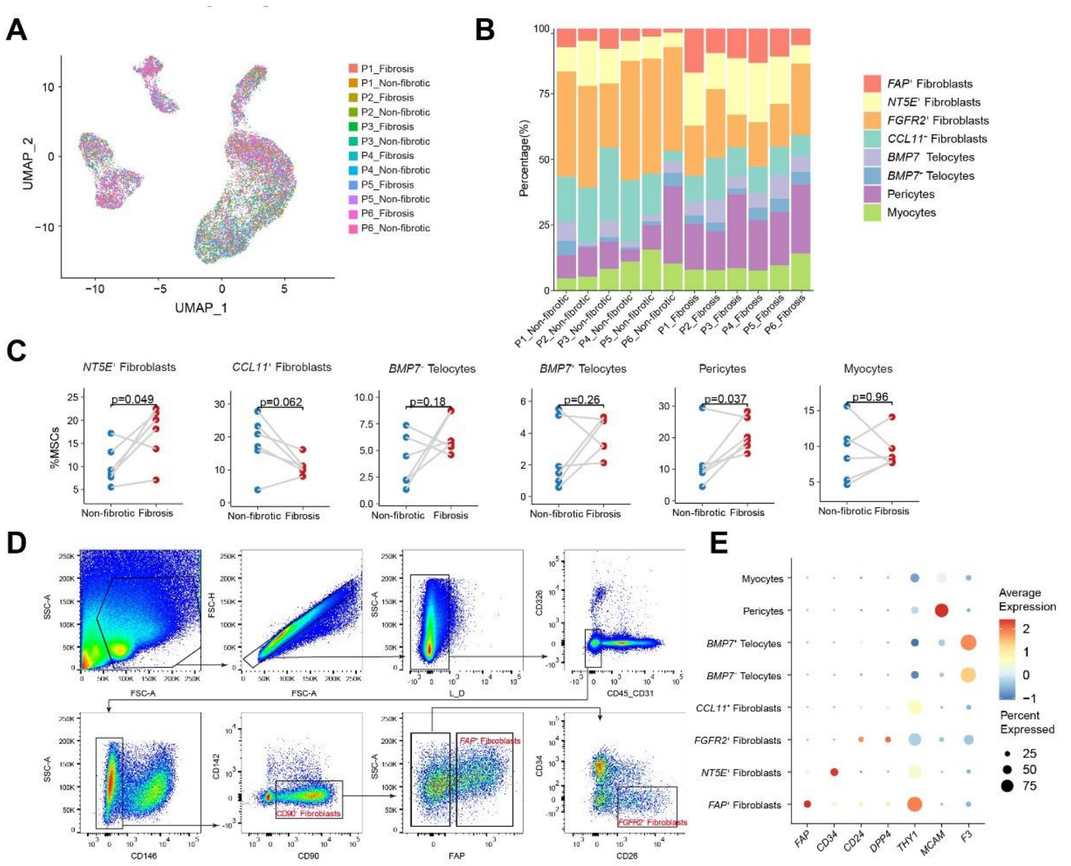

Supplemental Figure 2. Heterogeneity of the mesenchymal stromal cells in intestinal fibrosis.

(**A**) UMAP plot showing MSCs distribution across 6 fibrotic and 6 non-fibrotic samples. R package harmony was used to correct batch effects and constructed one UMAP based on MSCs.

(**B**) Bar plots showing percentage of MSCs subclusters across 12 samples.

(**C**) Comparison of frequencies of MSCs subclusters in paired fibrotic intestinal samples (n = 6) and non-fibrotic intestinal samples (n = 6). Statistical difference were determined by paired t tests.

(**D**) Flow cytometry gating strategy for *FAP*$^{+}$ fibroblasts and *FGFR2*$^{+}$ fibroblasts.

(**E**) Dot plots of the markers used by flow cytometry gating in scRNA-seq data. The average gene expression and percentage of cells expressed are shown by dot colour and size, respectively.

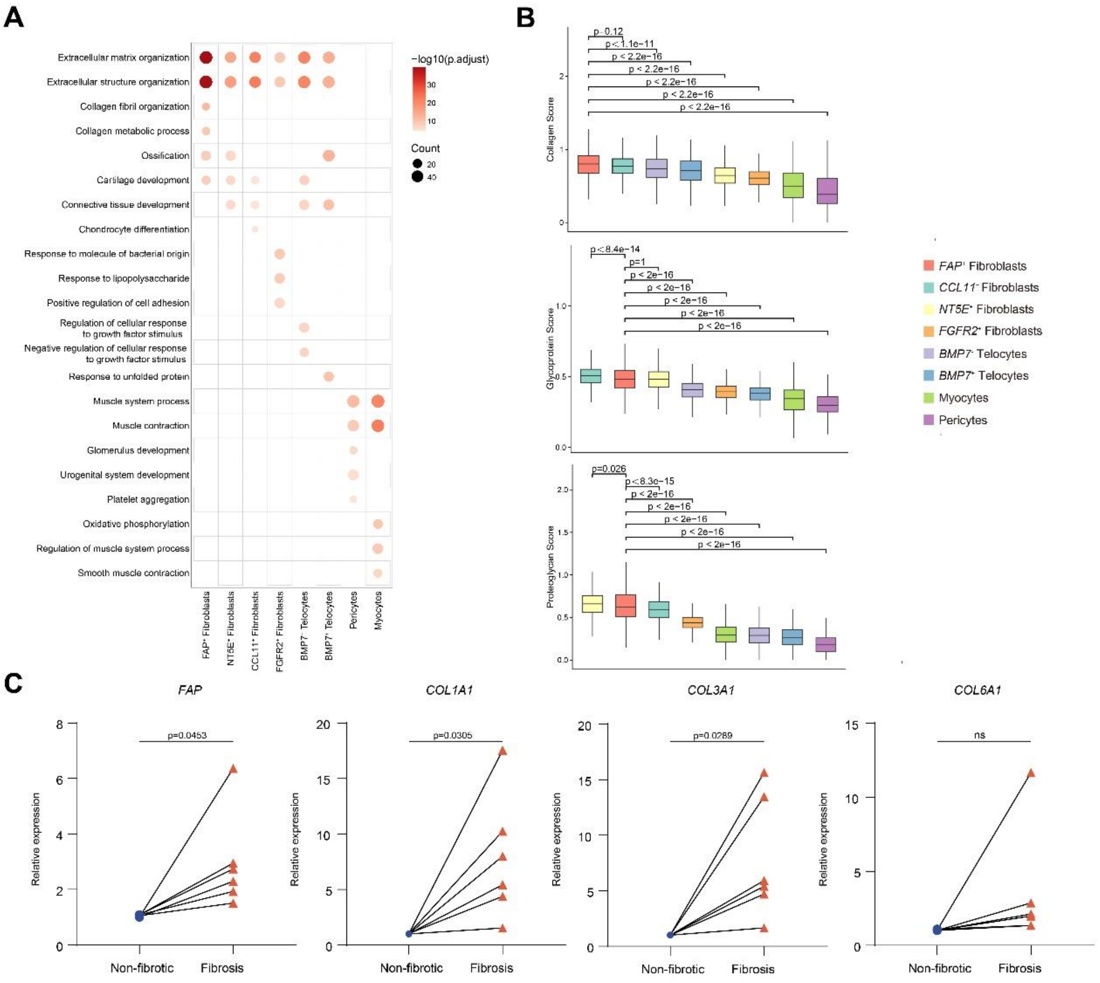

Supplemental Figure 3. Heterogeneity of the mesenchymal stromal cells in intestinal fibrosis.

(**A**) Dot plots showing top 5 significant gene ontology (GO) enrichment terms in all MSCs subclusters. A hypergeometric test was performed with FDR-adjusted P values.

(**B**) Boxplots showing respective ECM signature score (collagen, glycoprotein and proteoglycan) of each MSCs subcluster in fibrosis states. Statistical difference were determined by one-way ANOVA with Bonferroni correction.

(**C**) The relative expression of the ECM-related genes in the non-fibrotic and fibrotic intestinal tissues was analyzed by qPCR. The points corresponding to the paired samples (n=6) in the graph are connected. Statistical difference were determined by paired t tests.

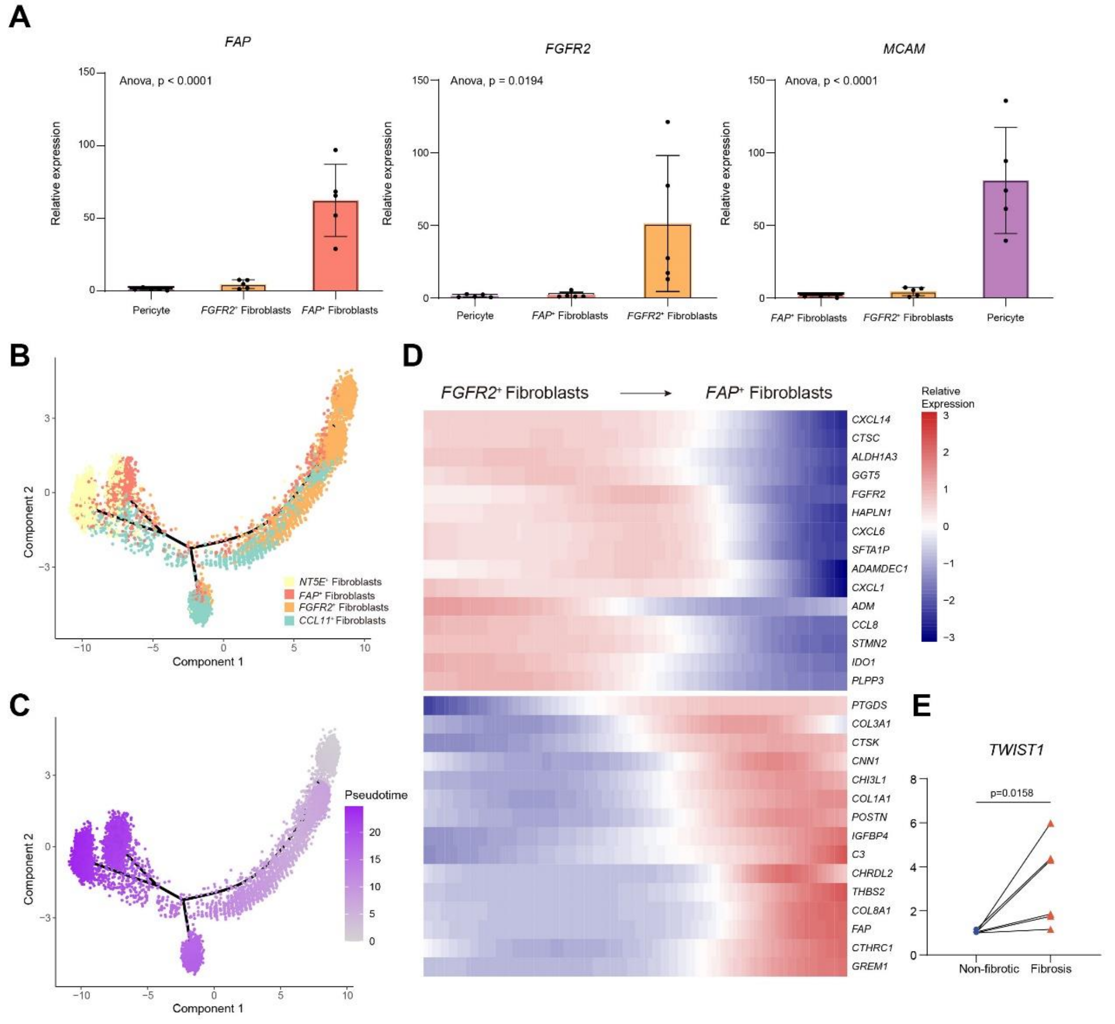

Supplemental Figure 4. TWIST1 is a critical transcription factor in the differentiation of *FAP*$^{+}$ fibroblasts.

(**A**) The mRNA levels of FAP, FGFR2 and MCAM in *FAP*$^{+}$ fibroblasts, *FGFR2*$^{+}$ fibroblasts and pericytes sorted from fibrotic sites(n=5) were analyzed by qPCR to verify the purity of sorted cells. Statistical difference were determined by one-way ANOVA.

(**B-C**) Inferred differentiation trajectory for 4 fibroblast subclusters by monocle. Each dot represents a cell, and colour represents the annoted subclusters (**B**) and estimated pseudotime for each cell (**C**).

(**D**) Heatmap showing the dynamic expression (z score) changes of representative genes along the differentiation pseudotime of *FGFR2*$^{+}$ fibroblasts to *FAP*$^{+}$ fibroblasts.

(**E**) The relative expression of TWIST1 in the non-fibrotic and fibrotic intestinal tissues was analyzed by qPCR. The points corresponding to the paired samples (n=6) in the graph are connected. Statistical difference was determined by paired t test.

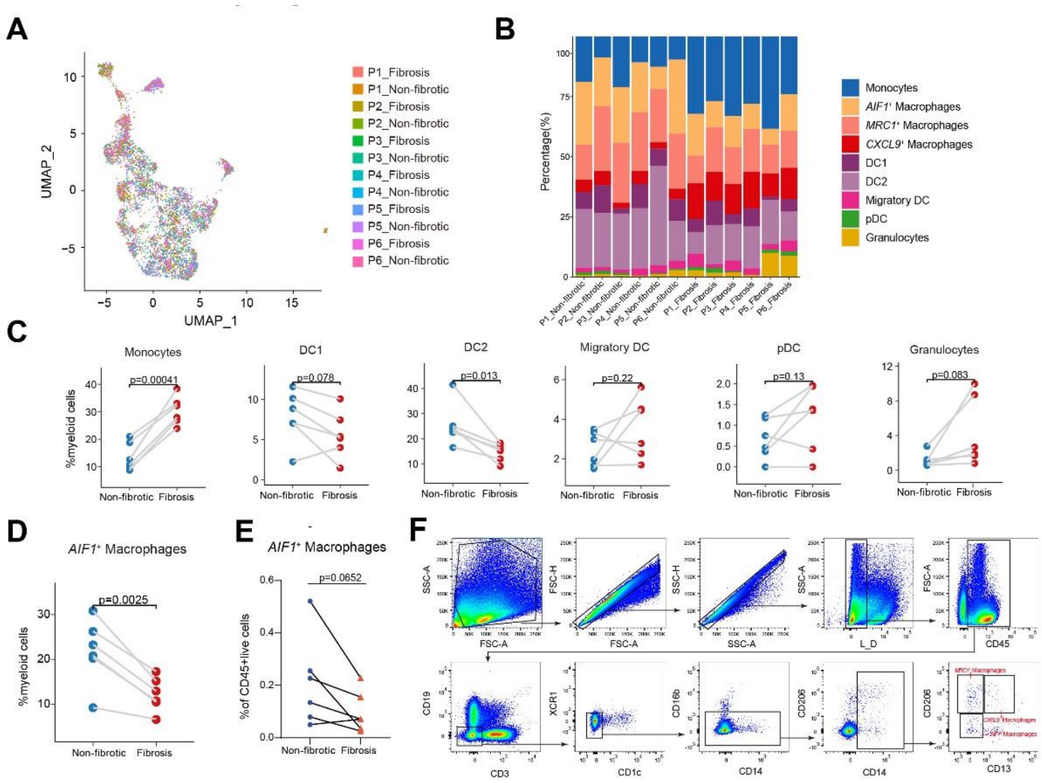

Supplemental Figure 5. Identification of profibrotic macrophage phenotypes and their interactions with *FAP*+ fibroblasts in intestinal fibrosis.

(**A**) UMAP plot showing myeloid cells distribution across 6 fibrotic and 6 non-fibrotic samples. R package harmony was used to correct batch effects and constructed one UMAP based on myeloid cells.

(**B**) Bar plots showing percentage of myeloid cells subclusters across 12 samples.

(**C-D**) Comparison of frequencies of myeloid cells subclusters in paired fibrotic intestinal samples (n = 6) and non-fibrotic intestinal samples (n = 6). Statistical difference were determined by paired t tests.

(**E**) Flow cytometry analysis revealed the proportion variation of *AIF1*+ macrophages to CD45+ live cells in non-fibrotic and fibrotic sites. The points corresponding to the paired samples (n=6) in the graph are connected. Statistical difference was determined by paired t test.

(**F**) Flow cytometry gating strategy for myeloid cells.

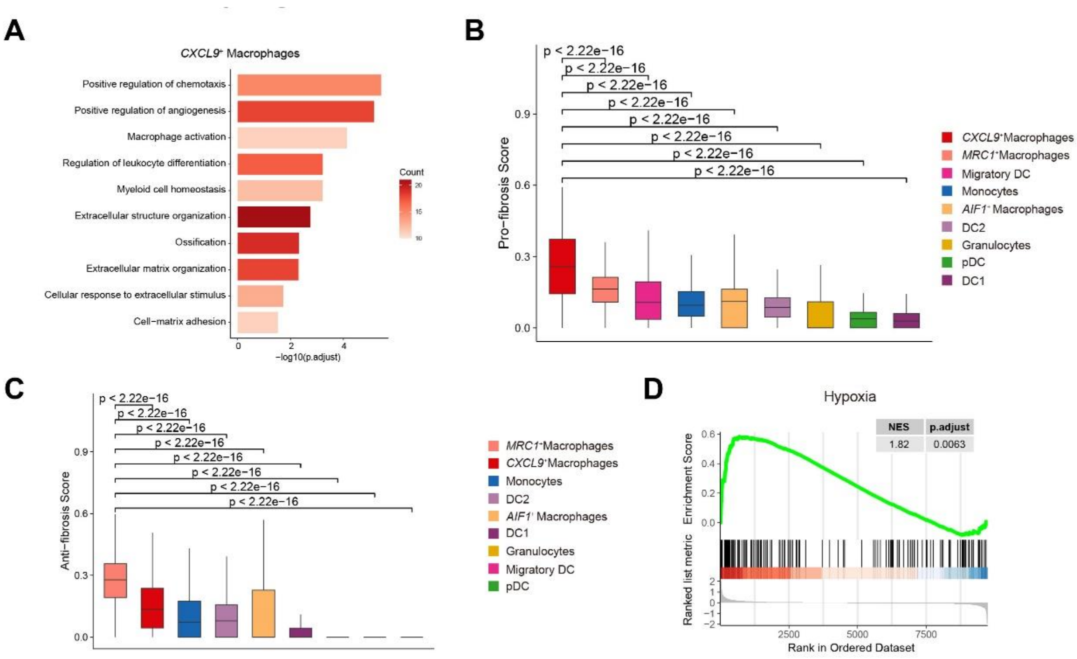

Supplemental Figure 6. Identification of profibrotic macrophage phenotypes and their interactions with *FAP*$^+$ fibroblasts in intestinal fibrosis.

(**A**) Representative gene ontology (GO) enrichment of the marker genes expressed in *CXCL9*$^+$ macrophages. A hypergeometric test was performed with FDR-adjusted P values.

(**B-C**) Boxplots showing profibrotic(**B**) and antifibrotic(**C**) signature score of each myeloid subcluster. Statistical differences were determined by one-way ANOVA with Bonferroni correction.

(**D**) Gene Set Enrichment Analysis (GSEA) of hypoxia gene sets from 50 hallmark gene sets in MSigDB between *CXCL9*$^+$ macrophages and the other two macrophage clusters. NES, normalization enrichment score.

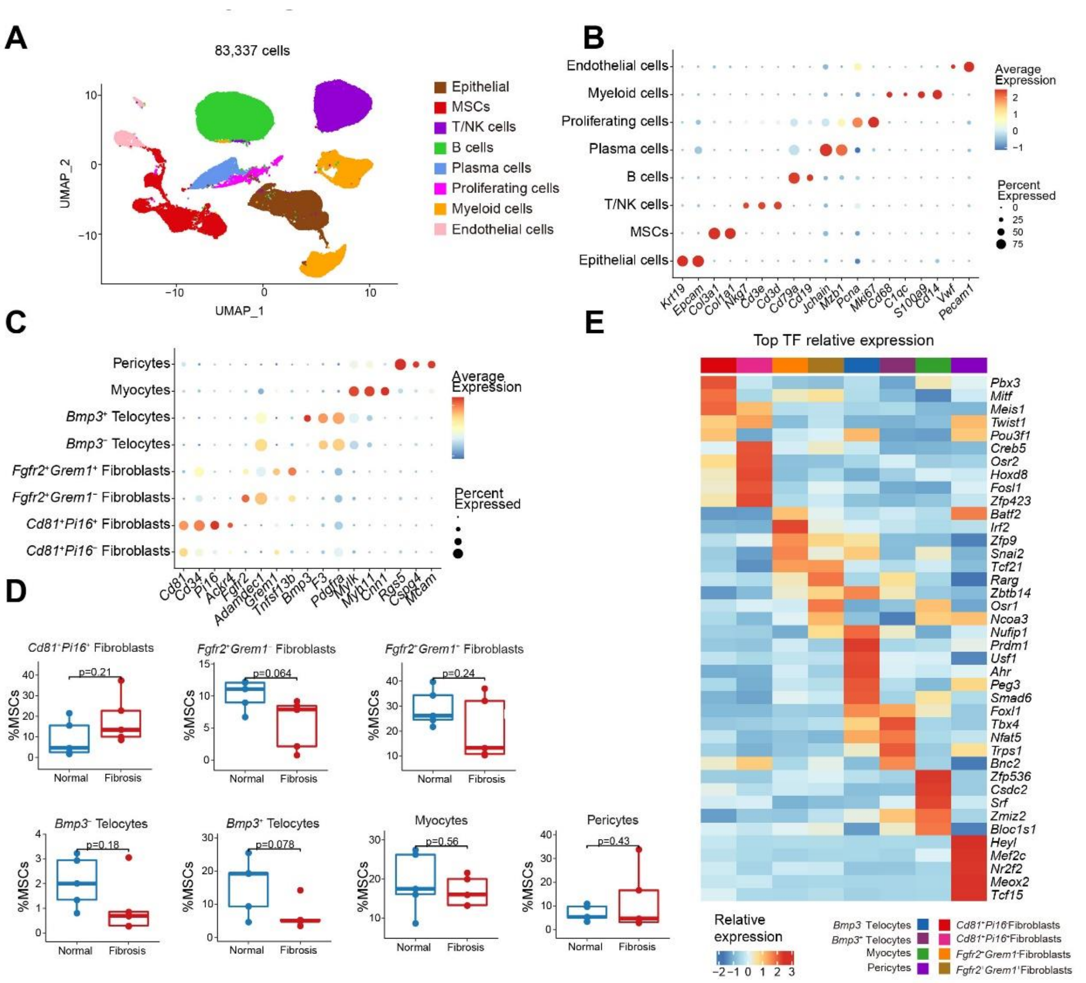

Supplemental Figure 7. Single-cell sequencing on DSS-induced fibrosis mouse model.

(**A**) UMAP plot of major cell types of mouse model.

(**B**) Dot plots of the representative markers of major cell types of mouse model. The average gene expression and percentage of cells expressed are shown by dot color and size, respectively.

(**C**) Dot plots of the representative markers of subclustered MSCs of the mouse model. The average gene expression and percentage of cells expressed are shown by dot color and size, respectively.

(**D**) Boxplots showing differential composition of MSCs subclusters between DSS-treated (n=5) and control mice (n=5). Statistical differences were determined by t tests.

(**E**) Heatmap showing the relative expression (z score) of top 5 TF genes in each MSCs subtypes.

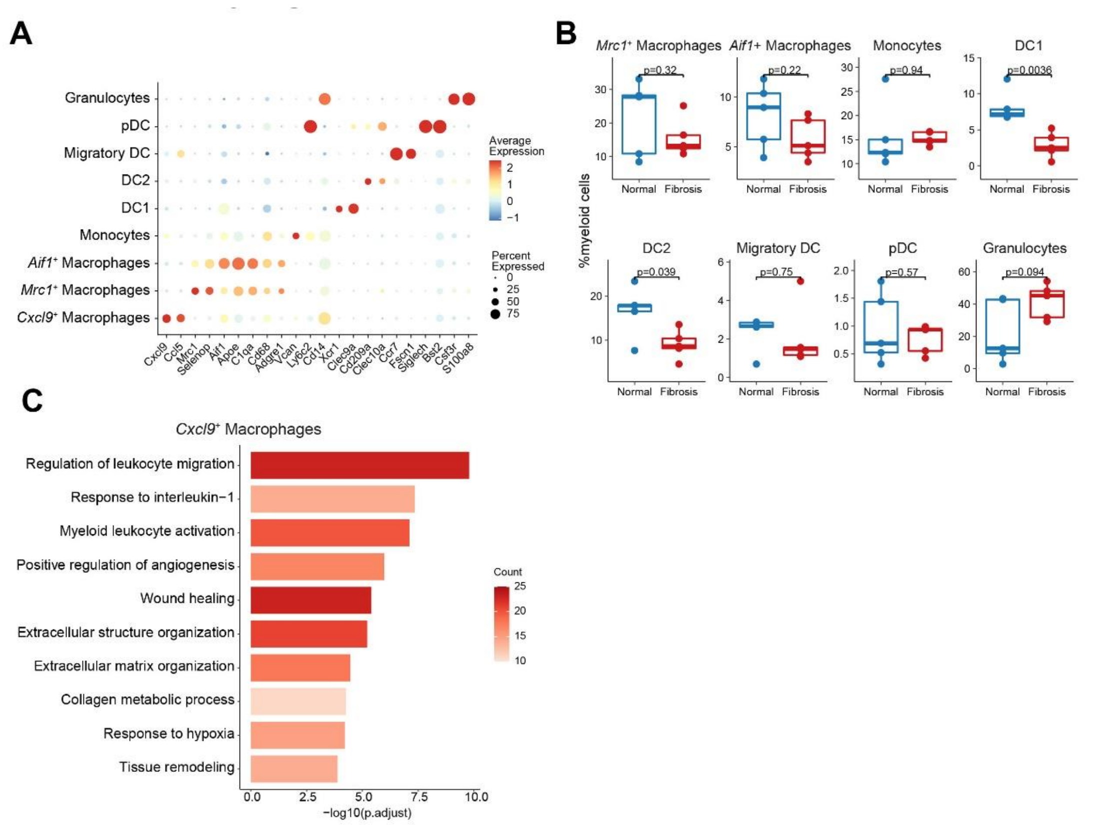

Supplemental Figure 8. Single-cell sequencing on DSS-induced fibrosis mouse model.

(**A**) Dot plots of the representative markers of subclustered myeloid cells in the mouse model. The average gene expression and percentage of cells expressed are shown by dot color and size, respectively.

(**B**) Boxplots showing differential composition of myeloid subclusters between DSS-treated (n=5) and control mice (n=5). Statistical differences were determined by t tests.

(**C**) Representative gene ontology (GO) enrichment of the marker genes expressed in *Cxcl9*$^{+}$ macrophages. A hypergeometric test was performed with FDR-adjusted P values.

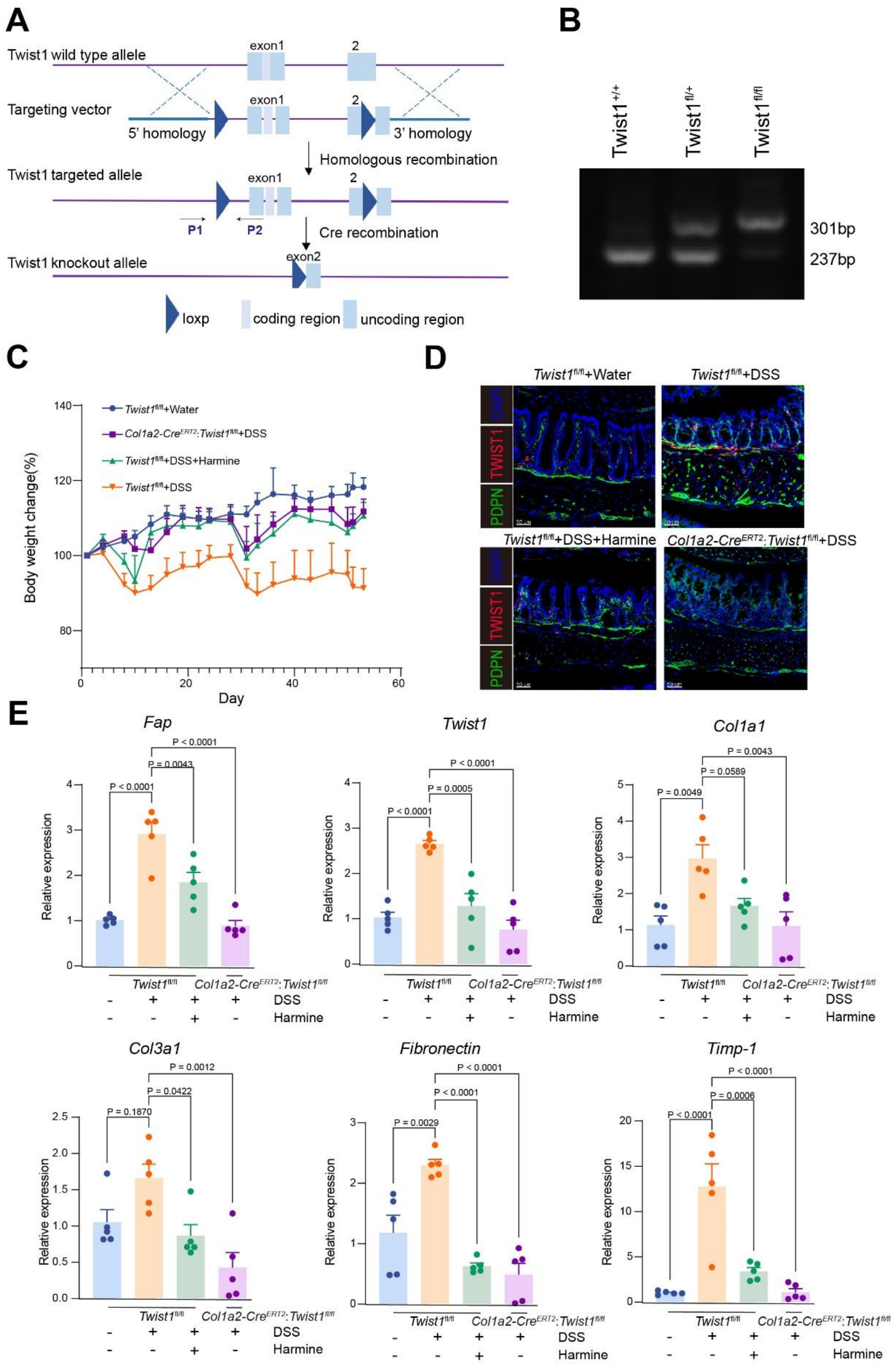

Supplemental Figure 9. Targeting TWIST1 inhibits fibroblast activation and attenuates experimental intestinal fibrosis.

(**A**) Schematic diagram showing the strategy for generation of Twist1$^{fl/fl}$ mice by homologous recombination using CRISPR/Cas9. Exon 1-2 of the transcript Twist1-201

was selected as the flox region. P1 and P2 are primers used for PCR to distinguish the wild type and Twist1$^{fl/fl}$ alleles.

(**B**) Genotyping of Twist1$^{fl/fl}$ mice with P1 and P2 primers. DNA was prepared from tails of Twist1$^{fl/fl}$ mice.

(**C**) The mouse weight change curve (normalized to the first day) of 4 indicated mouse groups.

(**D**) Representative IF staining of mouse colons among 4 indicated groups. (20x). DAPI (blue), TWIST1 (red) and PDPN (green). Merged channels are shown. Bar, 50 µm.

(**E**) The mRNA levels of fibrosis-related genes of 4 indicated mouse groups were analyzed by qPCR. Data represent mean ± SD. Statistical difference were determined by one-way ANOVA with Bonferroni correction.

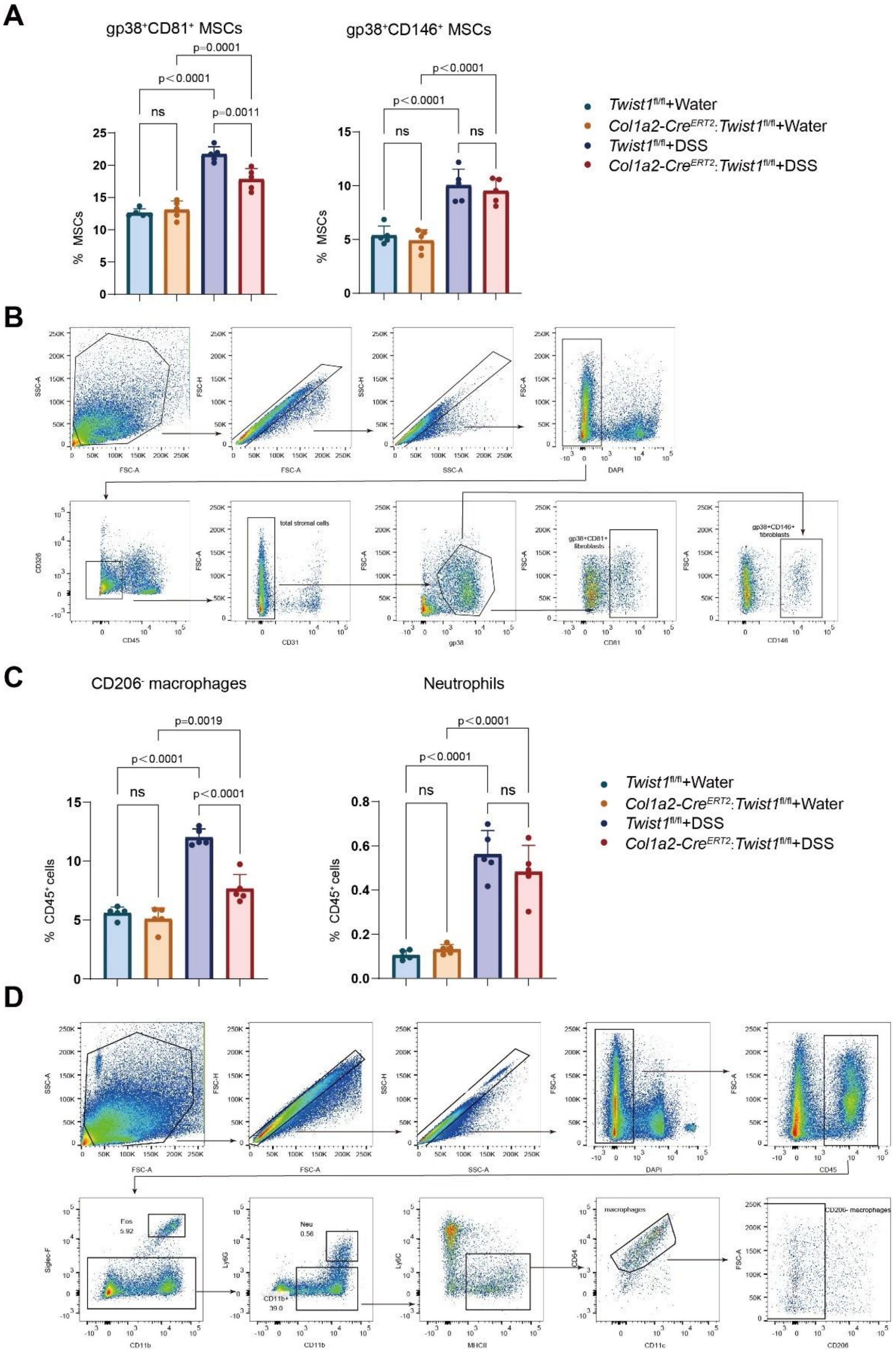

Supplemental Figure 10. Flow cytometry of MSCs and myeloid cell subsets in *Col1a2-Cre^{ERT2}:Twist1$^{fl/fl}$* mice.

(**A**) Bar plot showing the proportional variation in gp38^{+}CD81^{+} MSCs (left) and gp38^{+}CD146^{+} MSCs to total MSCs (CD326^{-}CD45^{-}CD31^{-}) in *Col1a2-Cre^{ERT2}:Twist1$^{fl/fl}$* and *Twist1$^{fl/fl}$* mice undergoing chronic DSS treated water control. Statistical differences

were determined by one-way ANOVA with Bonferroni correction. (n=5 per group)
(**B**) Flow cytometry gating strategy for mice MSCs.
(**C**) Bar plot showing the proportional variation in $CD206^-$ macrophages (left) and neutrophils to total $CD45^+$ cells in *Col1a2-Cre*^{ERT2}*:Twist1*$^{fl/fl}$ and *Twist1*$^{fl/fl}$ mice undergoing chronic DSS treated water control. Statistical differences were determined by one-way ANOVA with Bonferroni correction. (n=5 per group)
(**D**) Flow cytometry gating strategy for mice myeloid cells.

Supplemental tables:

Supplemental Table 1. Clinical information of patients included for scRNA-seq, related to Figure1 and Figure S1

Patient ID	P1	P2	P3	P4	P5	P6
**Age（y/o)**	44	28	44	27	51	52
**Gender**	Male	Male	Male	Female	Male	Female
**Disease duration (y)**	5	5	7	8	10	8
**Smoking status**	Never	Never	Never	Never	Never	Never
**BMI**	23.55	18.52	20.67	28.36	20.41	20.1
**Therapy at time of surgery**	None	Anti-TNFα	None	5-ASA	AZA	MTX
**Motreal classification**						
**Age at diagnosis**	A2	A2	A2	A2	A3	A3
**Location**	L1	L3	L1	L3	L1	L1
**Behaviour**	B2	B3	B2	B3	B2	B3
**Perianal disease**	No	No	No	No	No	No
**Stricture site**	ileum	ileum	ileum	ileum	ileum	ileum
**Creeping fat**	Yes	No	Yes	Yes	Yes	Yes

y/o, years old; y, years; 5-ASA, 5-Aminosalicylic acid; AZA, Azathioprine; MTX, Methotrexate; A1, below 16 y/o; A2, between 17 and 40 y/o; A3, above 40 y/o; L1, ileum; L2, colonic; L3, ileocolonic; L4, isolated upper disease; B1, non-stricturing, non-penetrating; B2, stricturing; B3, penetrating.

Supplemental Table 2. Primer sequences for qPT-PCR

Gene	Species	Forward primer	Reverse primer
COL1A1	Homo sapiens	GTGCGATGACGTGATCTGTGA	CGGTGGTTTCTTGGTCGGT
COL3A1	Homo sapiens	GCCAAATATGTGTCTGTGACTCA	GGGCGAGTAGGAGCAGTTG
COL6A1	Homo sapiens	ACAGTGACGAGGTGGAGATCA	GATAGCGCAGTCGGTGTAGG
ACTA2	Homo sapiens	GTGTTGCCCCTGAAGAGCAT	GCTGGGACATTGAAAGTCTCA
POSTN	Homo sapiens	GACCGTGTGCTTACACAAATTG	AAGTGACCGTCTCTTCCAAGG
TWIST1	Homo sapiens	AGCTACGCCTTCTCGGTCT	CCTTCTCTGGAAACAATGACATC
FAP	Homo sapiens	TGAACGAGTATGTTTGCAGTGG	GGTCTTTGGACAATCCCATGT
FGFR2	Homo sapiens	GGAAAGTGTGGTCCCATCTGA	TCCAGGTGGTACGTGTGATTG
MCAM	Homo sapiens	GAAGTCACCGTCCCTGTTTTC	CCCCGTTGTCGTTGGTTGT
GAPDH	Homo sapiens	ACAACTTTGGTATCGTGGAAGG	GCCATCACGCCACAGTTTC
Fap	Mus musculus	GTCACCTGATCGGCAATTTGT	CCCCATTCTGAAGGTCGTAGAT
Twist1	Mus musculus	GAGCAAGATTCAGACCCTCAA	CATCTTGGAGTCCAGCTCGT
Col1a1	Mus musculus	CTTCACCTACAGCACCCTTGTG	CTTGGTGGTTTTGTATTCGATGAC
Col3a1	Mus musculus	GAAAGAGGATCTGAGGGCTCG	GGGTGAAAAGCCACCAGACT
Fibronectin	Mus musculus	ATGTGGACCCCTCCTGATAGT	GCCCAGTGATTTCAGCAAAGG

Timp-1	Mus musculus	GCTTGGTTCCCTGGCGTACTCT	GTGAGTGTCACTCTCCAGTTTGC
Gapdh	Mus musculus	CAGTGGCAAAGTGGAGATTGTTG	CTCHCTCCTGGAAGATGGTGAT

Supplemental Table 3. Signature genes for each cell cluster (human).

**Cell types**	**Top marker genes**
MSCs	*LUM, DCN, PTGDS, CFD, COL3A1, COL1A2, ACTA2, COL1A1, IGFBP5, COL6A2*
Endothelial cells	*PECAM1, VWF, FABP5, ACKR1, RAMP2, SLC9A3R2, HYAL2, RAMP3, ID1, SOX18*
Myeloid cells	*IL1B, LYZ, S100A9, CXCL8, C1QA, CCL3, C1QB, HLA-DRA, HLA-DPA1, G0S2*
B cells	*MS4A1, CD79A, BANK1, CD37, HLA-DRA, CD83, HLA-DQA1, HLA-DQB1, CCR7, HLA-DPB1*
Plasma cells	*JCHAIN, IGKC, IGHA1, IGHA2, IGHG1, IGLC1, IGHG3, MZB1 ,SSR4, DERL3*
Glial cells	*CRYAB, S100B, GPM6B, NRXN1, PLP1, CDH19, SCN7A, PMP22, CLU, ALDH1A1*
Mast cells	*TPSB2, TPSAB1, CTSG, CPA3, GATA2, MS4A2, AREG, IL1RL1, ADCYAP1, HDC*
T/ILCs	*CD3D, CD3E, IL7R, CCL5, KLRB1, CD2, TRBC2, TRAC, IFNG, NKG7*
Epithelial cells	*KRT18, EPCAM, KRT19, MUC2, KRT20, FABP6, AGR2, KRT8, ALDOB, REG3A*
FAP+ Fibroblasts	*FAP, TWIST1, CHI3L1, CTHRC1, IGFBP4, GREM1, CTSK, THBS2, C3, FBLN2*
NT5E+ Fibroblasts	*NT5E, SFRP2, MGP, CCDC80, DPT, C7, RSPO3, GSN, OGN, CXCL12*
FGFR2+ Fibroblasts	*FGFR2, ADAMDEC1, CXCL14, CTSC, CXCL1, ALDH1A3, CCL8, CXCL6, CXCL8, HAPLN1*
CCL11+ Fibroblasts	*CCL11, CCL13, SFRP1, CFD, PTGD, ADAMDEC1, FABP5, FNDC1, TNXB, FBLN1*
BMP7+ Telocytes	*PDGFRA, F3, FOXL1, BMP7, NRG1, ID1, NPY, AGT, SOX6, TRPA*

BMP7- Telocytes	*PDGFRA, F3, FOXL1, ALKAL2, APO, EDNRB, PCSK6, LY6H, SOX6, MMP11*
Pericytes	*PDGFRB, MUSTN1, RGS5, TINAGL1, MT1M, RGS16, NOTCH3, NDUFA4L2, MCAM, CPE*
Myocytes	*DES, ACTG2, MYH11, HHIP, CKB, GREM2, CNN1, TPM2, FLNA, TAGLN*
Monocytes	*IL1B, IL1A, PTGS2, VCAN, FCN1, SOD2, NLRP3, IER3, OLR1, CD44*
CXCL9+ macrophages	*CXCL9, CHI3L1, CAPG, MMP9, CXCL10, IL4I1, IL32, NR1H3, LILRB4, BHLHE41*
MRC1+ macrophages	*MRC1, SELENOP, MAF, FOLR2, C1QA, A2M, IGF1, PLD3, CD163, CSF1R*
AIF1+ macrophages	*AIF1, TMSB4X, TPT1, HLA-DPA1, HLA-DPB1, CD74, TYROBP, HLA-DRA, APOE, B2M*
Granulocytes	*FCGR3B, CSF3R, S100A8, S100A9,CXCR1,CXCR2,IFITM2,SOD2,SRGN,CXCL8*
DC1	*CLEC9A,CADM1,CLNK,XCR1,ENPP1,TACSTD2,DBN1, ASB2,BTLA,CCND1*
DC2	*CD1C,FCER1A,CD207,AFF3,CD1E,CD52,PLD4,CD1D, NDRG2,PRMT9*
Migratory DC	*LAMP3,FSCN1,CCR7,LAD1,NCCRP1,CD200,TREML1, CCL19,INSM1,CCL22*
pDC	*CLEC4C,LILRA4,GZMB,CLIC3,SCT,TCL1A,MYBL2,SMPD3,LRRC26,MAP1A*

Supplemental Table 4. Signature genes for each cell cluster (mouse).

**Cell types**	**Top marker genes**
MSCs	*Dcn, Col3a1, Gsn, Col1a2, Col1a1, Dpt, Postn, Fn1, Igfbp4, Igfbp5*
Endothelial cells	*Pecam1, Vwf, Ccl21a, Fabp4, Flt1, Plvap, Ly6c1, Igfbp7, Podxl, Adgrf5*
Myeloid cells	*S100a9, S100a8, Cxcl2, Il1b, Lyz2, Tyrobp, Cd14, Clec4e, Ifitm1, Fcer1g*
B cells	*Cd79a, Ly6d, Ighm, Cd79b, Ebf1, Ms4a1, Scd1, Ighd, H2-Ob, Cd74*
Plasma cells	*Mzb1, Igha, Jchain, Iglv1, Iglv2, Iglc1, Iglc3, Iglc2, Xbp1, Igkc*
Proliferating cells	*Mki67, Stmn1, Pclaf, Hmgb2, Top2a, Tubb5, Ube2c, Tuba1b, Cks1b, Smc4*
T/NK cells	*Cd3d, Nkg7, Ccl5, Icos, Trbc2, Cd3e, Cd3g, Il7r, Gzmb, Trbc1*
Epithelial cells	*Epcam, Krt8, Saa1, Krt19, Muc3, Tff3, Muc2, Muc13, Cldn7, Tspan1*
Cd81+Pi16- Fibroblasts	*Cd81, Ptn, C3, Dcn, Smoc2, Il33, Rspo3, Thbs2, Ackr4, Fbln1*
Cd81+Pi16+ Fibroblasts	*Cd81, Pi16, Cd34, Col14a1, Igfbp6, Cilp, Pcolce2, Mfap5, Plxdc2, Dcn*
Fgfr2+Grem1- Fibroblasts	*Fgfr2, Adamdec1, Igfbp3, Col15a1, Col6a5, Igfbp4, Lpl, Ccl11, Nrp1, Fn1*
Fgfr2+Grem1+ Fibroblasts	*Fgfr2, Grem1, Eln, Sfrp2, Mgp, Igfbp4, Tnfsf13b, Gsn, Cd55, Cxcl12*
Bmp3- Telocytes	*Pdgfra, Foxl1, Procr, F3, Bmp2, Nrg1, Ptprr, Glp2r, Ptpre, Il1rl1*
Bmp3+ Telocytes	*Pdgfra, Foxl1, Procr, F3, Bmp3, Wnt5a, Tcf4, Nbl1, Sox6, Adam19*

Pericytes	*Rgs5, Notch3, Mcam, Pdgfrb, Rgs4, Ebf1, Rasd1, Myo1b, Esam, Heyl*
Myocytes	*Hhip, Myh11, Acta2, Actg2, Tagln, Myl9, Mylk, Tpm2, Cnn1, Lmod1*
Monocytes	*Ly6c2, Cd14, S100a4, Lyz2, Vcan, Ly6e, Ccr2, Ms4a4c, Tmsb10, Npc2*
Cxcl9+ macrophages	*Cxcl9, Cd68, Ccl5, Cxcl10, Nos2, Sdc4, Mmp14, Il1a, Ly6i, Nrg1*
Mrc1+ macrophages	*Mrc1, Selenop, Folr2, C1qb, C1qc, C1qa, Ccl8, Ccl7, Apoe, Fcrls*
Aif1+ macrophages	*Aif1, H2-Aa, H2-Ab1, H2-Eb1, C1qa, C1qb, C1qc, Mmp13, Itga9, Mmp14*
Granulocytes	*Csf3r, S100a8, S100a9, Retnlg, Wfdc21, G0s2, Cstdc4, Pglyrp1, Lcn2, Hdc*
DC1	*Clec9a, Wdfy4, Xcr1, Itgae, Ears2, Flt3, Ncoa7, Hic1, Lrrk2, H2-Ob*
DC2	*Cd209a, Klrd1, H2-DMb2, Cd209c, H2-Oa, Klrb1b, Ramp3, Kit, Clec10a, Ctnnd2*
Migratory DC	*Ccr7, Fscn1, Il12b, Ccl22, Il4i1, Socs2, Cd200, Sema7a, Ccser2, Mreg*
pDC	*Ly6d, Siglech, Bst2, Ccr9, Cd8b1, Cd7, Lrp8, Atp1b1, Spib, Cd300c*

Supplemental Table 5. Signature genes used to define functional genesets.

Signature	Genes
**ECM**	*ITGA2, COL5A1, LAMC3, DCN, LAMA3, CPB2, ITGA11, PRSS2, CYP1B1, COL6A3, COL6A2, COL6A1, COL11A1, FSCN1, CTRB2, HSPG2, FAP, PLOD3, GREM1, COL4A1, COL3A1, COL2A1, COL1A1, FERMT1, NPHS1, LAMA2, THBS1, LAMB1, VCAM1, BCAN, CTRB1, TNXA, ABL1, BCL3, DPP4, PRDX4, OLFML2A, SPARC, FN1, CCDC80, MMP11, LOXL1, DDR1, CAPN2, TLL1, CAPN1, MPV17, ANXA2, PDGFA, CMA1, VTN, CTSV, MATN3, LAMA5, ITGA10, COL8A2, COL18A1, ADAMTS20, ADAM19, LAMA4, RECK, COL5A2, MMP20, SPINK5, RIC8A, DDR2, SPINT2, COMP, CTGF, CLASP2, SERAC1, FLOT1, CARMIL2, ICAM5, ENG, ATXN1L, MMP8, SERPINE1, FMOD, NID2, FGF2, ANTXR1, VWA1, THSD4, TNXB, ITGAX, ITGAL, SPP1, COL19A1, GFOD2, ATP7A, NOXO1, DNAJB6, ADAMTS4, ADAM12, FGFR4, MATN4, ADAMTS2, COL5A3, IHH, ERO1A, SFRP2, ELN, CTSS, KDR, KLK2, SMOC2, NDNF, FBLN5, VPS33B, OPTC, SMAD3, SPINT1, PLG, PRSS1, NR2E1, CREB3L1, ICAM4, ITGB1, MMP14, LRP1, P4HA1, COL7A1, ITGA4, VCAN, ADAM10, ABI3BP, DPT, MELTF, DSPP, CD47, NOX1, COL8A1, HSD17B12, PTK2, CRISPLD2, PDGFB, LOX, HAS2, MMP19, COL1A2, SPOCK2, COL16A1, ITGA8, IBSP, BGN, SCUBE3, TCF15, SERPINH1, TTR, CD44, JAM2, LCP1, VIPAS39, CFLAR, GAS6, ITGB2, MMP1, KLKB1, APP, FOXC1, B4GALT1, MYH11, PHLDB2, LAMB2, NFKB2, CTSG, ACAN, ITGB6, LAMB3, ITGA9, TGFB2, ELANE, NPHP3, CLASP1, MFAP2, FBLN1, KIF9, PECAM1, MATN1, ITGAM, HPN, VWF, MMP12, COL12A1, FOXC2, HPSE2, A2M, TPSAB1, F11R, COL13A1, HAS1, CTSK, ITGAE, APBB2, LOXL2, ITGB5, ERCC2, MPZL3, SH3PXD2A, NF1, COL4A4, C6orf15, COL9A1, CST3, TIMP1, AGT, ADAMTS14, LAMC1, TGFBI, POMT1, ITGAV, SOX9, CYR61, EGFLAM, TLL2, SERPINB5, ADAMTSL4, EXOC8, PDPN, CSGALNACT1, TNFRSF11B, PDGFRA, HTRA1, MMP16, MMP15, LOXL3, COL10A1, POSTN, ETS1, TIMP2, CAPNS1, SCX, TMPRSS6, ITGA7, CHADL, NOTCH1, MYF5, ITGB8, ITGB7, ADAMTSL2, ITGA3, FURIN, WNT3A, TNC, KAZALD1, LUM, NPNT, VIT, TNF, COL27A1, WASHC1, TGFBR1, AGRN, ADAM8, NID1, SH3PXD2B, GPM6B, MADCAM1, ADAM15, JAM3, TNR, COL14A1, ERO1B, TGFB1, DAG1, RAMP2, ITGB3, BSG, MYO1E, FOXF2, FOXF1, LAMA1, COL11A2, COL9A2, EGFL6, LAMC2, PHLDB1, ITGA2B, ELF3, MMP9, COL4A2, MMP2, MMP3, HAPLN1, MFAP4, SCUBE1, ECM2, SULF1, SULF2, HAS3, ITGB4, ITGA6, PXDN, MMP13, ICAM2, NCAN, COL9A3, COL4A6, FBN2, FBN1, HAPLN2, BMP1, MFAP5, SERPINF2, ITGA5, COL4A5, ICAM3, CTSL, FLRT2, KLK7, ICAM1, CAPNS2, ADAMTS5, RGCC, ITGAD, COL4A3, DMP1, FGG, FGB, FGA, ITGA1, ADAMTS3, CDH1, WT1, MMP10, MMP7*

**Collagen**	*COL10A1, COL12A1, COL14A1, COL16A1, COL1A1, COL1A2, COL21A1, COL3A1, COL5A1, COL5A2, COL6A2, COL6A3, COL8A1, COL8A2, COL11A2, COL13A1, COL15A1, COL17A1, COL23A1, COL25A1, COL4A1, COL4A2, COL22A1, COL24A1, COL26A1, COL4A6, COL9A2, COL18A1, COL19A1, COL27A1, COL4A3, COL4A4, COL4A5, COL6A1, COL7A1, COL28A1, COL5A3, COL9A3*
**Glycoprotein**	*EDIL3, IGFBP1, IGFBP2, NDNF, NPNT, NTNG1, PCOLCE2, SBSPON, SMOC1, WISP3, AGRN, BMPER, COCH, CRISPLD1, EMILIN2, FBLN2, FBN2, FBN3, FGB, FGG, FGL2, FRAS1, GLDN, HMCN1, IGFALS, LAMA1, LAMA3, LAMB1, LAMB3, LAMB4, LAMC2, LGI1, LGI2, LGI4, MATN4, MMRN1, MXRA5, NELL1, NELL2, NTN1, PAPLN, RELN, SLIT2, SPP1, TGFBI, TINAG, VWA5A, VWA5B1, VWA7, VWDE, ABI3BP, AEBP1, CILP, COMP, CRISPLD2, CTGF, CTHRC1, CYR61, DPT, ECM1, ECM2, EFEMP1, EFEMP2, EGFLAM, ELN, EMILIN1, FBLN1, FBLN5, FBLN7, FBN1, FNDC1, IGFBP3, IGFBP6, IGSF10, LAMA2, LAMA4, LAMC3, LTBP1, LTBP2, LTBP4, MATN2, MATN3, MFAP2, MFAP4, MFGE8, MGP, NID1, PCOLCE, POSTN, RSPO3, SLIT3, SMOC2, SPON1, SPON2, SRPX, SRPX2, SVEP1, THBS1, THBS2, THBS3, THBS4, THSD4, TNC, TNFAIP6, TSKU, VTN, WISP1, WISP2, BGLAP, COLQ, CRELD1, CRELD2, CRIM1, EMID1, EYS, FN1, GAS6, HMCN2, IGFBP4, IGFBP5, IGFBP7, KCP, LAMA5, LAMB2, LAMC1, LRG1, LTBP3, MFAP1, MFAP3, MFAP5, MMRN2, NID2, NOV, NTN4, NTN5, NTNG2, POMZP3, PXDN, SNED1, SPARC, SPARCL1, SSPO, TECTA, TINAGL1, TNXB, VWA1, VWCE, VWF, ZP3*
**Proteoglycan**	*CHADL, ESM1, HSPG2, IMPG2, PRG2, SRGN, ACAN, BGN, HAPLN2, HAPLN3, ASPN, DCN, FMOD, LUM, OGN, OMD, PODN, PODNL1, PRELP, VCAN, PRG4, SPOCK1, SPOCK2*
**Pro-fibrosis**	*LIPA, LPL, FDX1, SPP1, SPARC, MATK, GPC4, PALLD, CHI3L1, CHIT1, CTSK, MMP9, MMP7, CSF1, FCMR, TIMP3, SIGLEC15, CCL22*
**Anti-fibrosis**	*MMP1, MMP2, MMP14, MMP13, ITGA2, MRC1, MRC2, MFGE8*

Supplemental Table 6. Primer sequences for mice Genomic PCR

Primer	Sequence 5' → 3'	Primer type
P1	GGGGAATCCCTTGGGACTAGA	Forward
P2	CTGGGTCGCTGTTGCAGTC	Reverse
