# Supplementary material for: TWIST1+FAP+ fibroblasts in the pathogenesis of intestinal fibrosis in Crohn’s disease
Source: J Clin Invest. 2024 Jul 18;134(18):e179472. doi: 10.1172/JCI179472 (PMC11405050; doi:10.1172/JCI179472)

Full unedited gel for Figure7A

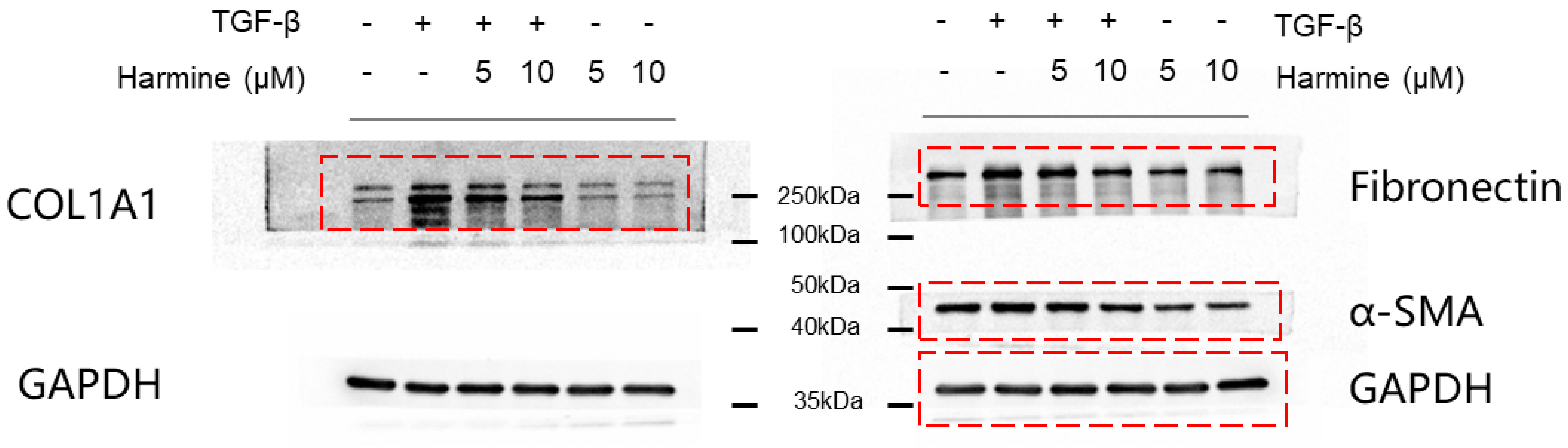

Full unedited gel for Figure7G

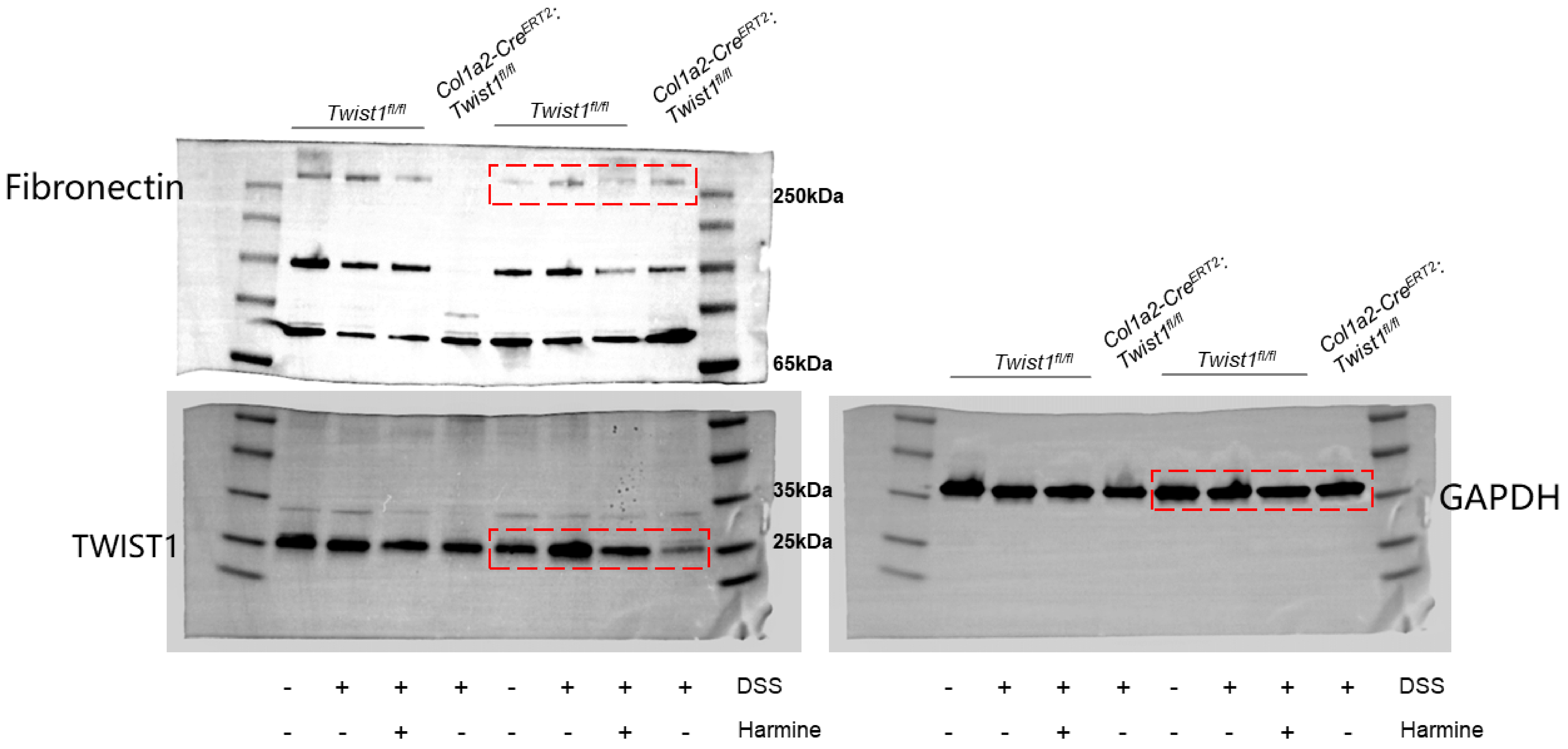

Full unedited gel for Supplemental Figure9B

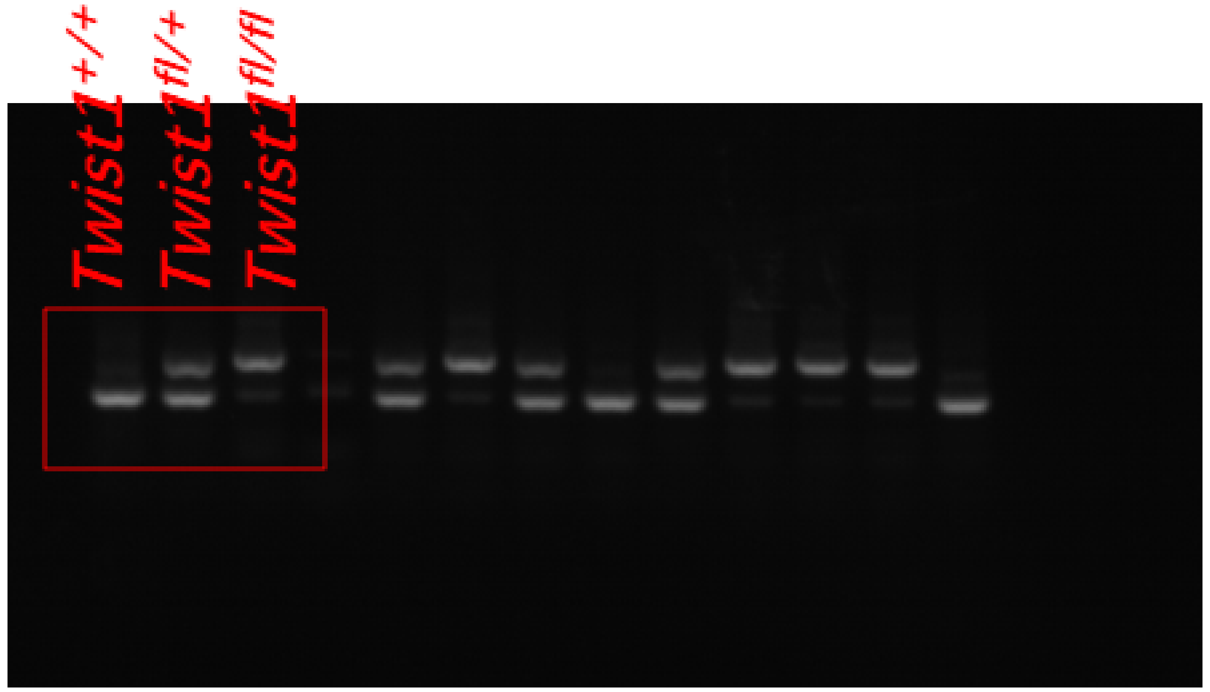

Supplement: Unedited blot and gel images [file jci-134-179472-s183.pdf]
